# Infectious complications following probiotic ingestion: a potentially underestimated problem? A systematic review of reports and case series

**DOI:** 10.1186/s12906-018-2394-3

**Published:** 2018-12-12

**Authors:** Rafael Lessa Costa, José Moreira, Andrea Lorenzo, Cristiane C. Lamas

**Affiliations:** 1 Intensive Care Unit of Unimed-Rio Hospital, Av. Ayrton Senna, 2550, Rio de Janeiro/RJ, CEP 22775-003 Brazil; 2 Cardiointensive Unit of Americas Medical City, Rio de Janeiro/RJ, Brazil; 3 National Institute of Cardiology, Rio de Janeiro/RJ, Brazil; 4 Unigranrio University, Rio de Janeiro/RJ, Brazil; 5 National Institute of Infectology Evandro Chagas, Fiocruz, Rio de Janeiro/RJ, Brazil

**Keywords:** Probiotics, Infection, Fungemia, Sepsis, *Saccharomyces*, *Lactobacillus*

## Abstract

**Background:**

Little is studied about complications related to probiotic ingestion. This study proposes to present a synthesis and critical evaluation of the reports and series of cases on the infectious complications related to the ingestion of probiotics, which can raise awareness for the prescribing and use of probiotics for certain groups of patients.

**Methods:**

Systematic review of reports and series of cases researched in the PubMed, SciELO and Scopus databases published until August 2018. The references of the articles were investigated manually for the search of cross references. SPSS version 23.0 was used for descriptive statistics and univariate analysis.

**Results:**

We found 60 case reports and 7 case series, making up a total of 93 patients. Fungemia was the most common infectious complications with 35 (37.6%) cases. The genus *Saccharomyces* was the most frequent with 47 (50.6%) cases, followed by *Lactobacillus*, *Bifidobacterium*, *Bacillus*, *Pedioccocus* and *Escherichia* with 26 (27.9%), 12 (12.8%), 5 (5.4%), 2 (2.2%) and 1 (1.1%) case, respectively. Adults over 60 years of age, *Clostridium difficile* colitis, antibiotic use and *Saccharomyces* infections were associated with overall mortality. HIV infections, immunosuppressive drugs, solid organ transplantation, deep intravenous lines, enteral or parenteral nutrition were not associated with death.

**Conclusion:**

The use of probiotics cannot be considered risk-free and should be carefully evaluated for some patient groups.

**Trial registration:**

CRD42016042289

**Electronic supplementary material:**

The online version of this article (10.1186/s12906-018-2394-3) contains supplementary material, which is available to authorized users.

## Background

The most commonly used and accepted definition of probiotics defines them as “live microorganisms, which are administered in adequate quantity and that confer benefits to the host’s health” [1].

In the last decades, several studies have demonstrated the benefit of probiotics for various diseases, including critically ill patients, in systematic reviews or meta-analyses [2–6].

In parallel, in some countries such as Finland, there has been a significant increase in the consumption of probiotics - from 1 l per person per year of *Lactobacillus* GG to 6 l per person per year [7].

According to the *Southern California Evidence-Based Practice Center*, infectious complications related to the use of probiotics are infrequently assessed in the outcomes and follow-ups of randomized clinical trials and when assessments are made, these are inconsistent. Therefore, current literature is not able to answer questions about the safety of probiotics in intervention studies [8].

Our study is a systematic review of case reports and case series evaluating serious infections such as bacteremia, fungemia, sepsis, endocarditis, abscess and other complications in the context of probiotic ingestion. We believe it may bring new evidence to clinical practice and possibly a more conscientious prescribing practice regarding antibiotics and probiotics.

Therefore, this systematic review aims to evaluate the most frequently reported infectious complications associated with the use of probiotics; the genera of probiotic microorganisms most frequently associated with infectious complications; populations and specific conditions most frequently affected with infectious complications; and finally, the factors associated with all-cause mortality among the reported cases.

## Methods

The description of this systematic review was based on the Preferred Reporting Items for Systematic Reviews and Meta-Analyzes – PRISMA [9]. The study was registered at PROSPERO (CRD42016042289).

### Inclusion criteria

Case reports or case series of patients were included, regardless of health status, age, previous use of probiotics, medication vehicle, presentation or microorganism involved, associated with infection in any system of the human body, with emphasis on the following diagnoses, according to their respective definitions or criteria: (1) bacteremia and fungemia, where microorganisms (bacteria or fungi) were isolated in blood [10], (2) sepsis, meaning infection related to the systemic inflammatory response according to the Sepsis Consensus Conference of 1992 [11], (3) endocarditis according to the Duke criteria [12], (4) abscess, when accumulation of purulent material in circumscribed tissues, organs or spaces, associated with signs of infection [13].

### Strategies for the search of studies and eligibility criteria

The searches and the review process were carried out in a restricted way to the reports or series of cases. The following electronic databases were searched: PubMed, Scielo and Scopus. For each base, search strategies were developed combining the following descriptors: “probiotics”, “*Lactobacillus*”, “*Bifidobacterium*”, “*Saccharomyces*”, “yogurt” with “sepsis”, “endocarditis”, “bacteremia”, “fungemia”, “abscess” and “infection” (see Additional file 1). There was no language restriction. The searches included papers from inception to August 8th 2018. Reference lists of all articles were manually searched for cross references. An electronic search database was created with the help of the Microsoft Excel 2011® program. Duplicate citations were deleted. Potentially relevant titles and abstracts were selected and their articles evaluated independently by Rafael Lessa da Costa and Jose Moreira. The divergences were resolved by consensus and, whenever necessary, by a third reviewer (Cristiane Lamas or Andrea De Lorenzo).

### Data extraction

The following key information was extracted from the selected studies (see Additional file 1):

- Publication data: title and authors of the article, name and year of the journal, volume, number and pages of the articles and contact email or address of the authors.
- Patient information: country of origin, age and gender, morbidity (prematurity, human immunodeficiency virus (HIV) / acquired immunodeficiency syndrome (AIDS), solid organ transplantation, immunosuppression, patient age, use of enteral or parenteral nutrition, presence of *Clostridium difficile* colitis, central venous access, prior diseases), time to onset of symptoms, microorganism involved and its sensitivity profile, type of biological sample from where the microorganism was isolated, method of comparison between the microorganism isolated in biological material and the probiotic used, and duration of treatment, type of infection and outcome (dead or alive). At this stage, the divergences were resolved by consensus and, when necessary, by the third reviewer. The authors were contacted to request necessary data not contained in the published version of the articles.

### Risk of bias assessment

A modified National Institutes of Health Tool [14] was to use to evaluate the risk of bias in the retrieved articles (see Additional file 1). In general terms, a ‘good’ study has the least risk of bias and the results are considered valid. A ‘fair’ study is susceptible to some bias considered insufficient to evaluate its results. A classification as ‘poor’ indicates a significant risk of bias.

### Statistical analysis

The patients were divided into groups according to outcome (dead or alive). Gender, age, preterm birth, *C. difficile* diarrhea, antimicrobial therapy duration, the etiology of the infectious complication, symptom onset time and duration of treatment were used for comparisons between groups. Categorical variables were expressed as counts and proportions, while continuous variables were expressed as mean and standard deviation or median and interquartile range, depending on the distribution of data. The categorical variables were compared with the *chi-square* test and Fisher’s exact test. Continuous variables were compared with the *Mann-Whitney* test. All tests used a two-tailed *p* value and < 0.05 was the cut-off point used for significance. SPSS version 23.0 (IBM, 2015) was used for statistical analysis.

## Results

### Database search

**Fig. 1:**
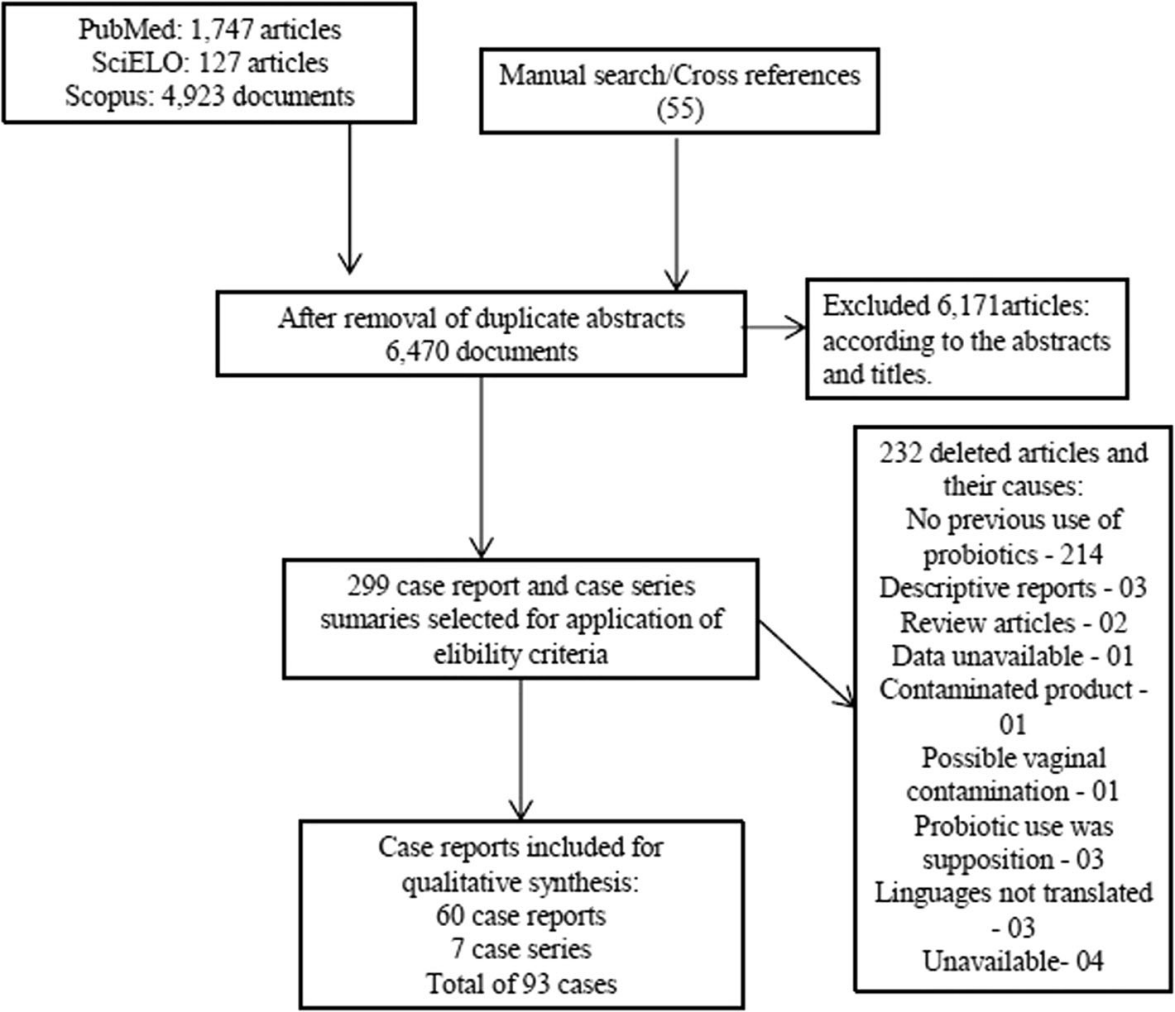
Flow diagram of the article selection process at each stage of the systematic review

As shown in the PRISMA flowchart (Fig. 1), searches in the three databases initially provided 6797 files. Another 55 titles were inserted through cross-references. After revision of the abstract and full-text articles, we ended up with 67 articles.We included 60 case reports and 7 case series, corresponding to 93 patients (Additional file 1).

### Geographical distribution of the manuscripts

Of the 60 reports and 7 series of cases involving 93 patients, most were from Europe (61; 65.6%) and 17 (18.3%) from the United States and Canada. Each of the five continents had at least one case. The earliest case dated back to 1976, and we observed a progressive increase in the following decades of 80, 90 and 00, with 4,16 and 33 cases, respectively. Between 2011 and 2017, there were 39 cases.

### Risk of bias assessment

Of the 67 articles included in the synthesis, 7 (10.4%) were classified as “good” and the remaining as “fair”. None were classified as “poor”.

### Occurrence of infectious complications

Fungemia was most commonly observed condition, with 35 (37.6%) of the cases. Sepsis was identified in 29 (31.2%) patients; bacteremia was responsible for 19 (20.4%) of cases, followed by endocarditis and abscess, with 4 and 3 cases, respectively. Pneumonia, pleural empyema and septic arthritis were present in only one case each.

### Probiotic microorganisms involved in infectious complications

The probiotic microorganisms involved in infectious complications are presented in Table 1. Details of each case are presented in Additional file 1: Tables S1-S5.

**Table 1:** Etiology of infectious complications after use of probiotics in 93 patients identified by systematic review, 1976–2018

Probiotics microorganisms	Absolute value (%)
*Saccharomyces cerevisiae*	41 (44.1)
*Saccharomyces boulardii*	5 (5.4)
*Saccharomyces spp.*	1 (1.1)
*Lactobacillus rhamnosus*	14 (15)
*Lactobacillus acidophilus*	4 (4.3)
*Lactobacillus spp.*	4 (4.3)
*Lactobacillus casei*	1 (1.1)
*Lactobacillus paracasei*	3 (3.2)
*Bifidobacterium longum*	6 (6.4)
*Bifidobacterium infantis*	3 (3.2)
*Bifidobacterium breve*	2 (2.1)
*Bifidobacterium spp.*	1 (1.1)
*Bacillus subtilis*	5 (5.4)
*Pediococcus pentosaceus*	1 (1.1)
*Pediococcus spp.*	1 (1.1)
*Escherichia coli*	1 (1.1)
Total	93 (100)

#### *Saccharomyces spp*.

Among the 93 cases, 47 (50.5%) were due to *Saccharomyces*, making it the most frequent genus in this review. In 5 patients, *Saccharomyces boulardii* (*S. boulardii*) was isolated from blood, in 41 cases *S. cerevisiae* was isolated from biological samples and in only one case the species was not identified. In 27 cases, the probiotic microorganisms in the medications were compatible with the isolates found in the blood samples of the patient. Antimicrobial susceptibility was available for only 12 of the 47 cases (Additional file 1: Table S6). All-cause mortality was 15% (14 cases).

#### *Lactobacillus spp*.

*Lactobacillus* spp. were the etiologic agents in 26 episodes of infectious complications after probiotic use; 14 were identified as *L. rhamnosus*, 4 as *L. acidophilus*, 3 as *L. paracasei*, 1 as *L. casei* and 4 cases were not identified at species level. Regarding gender, men comprised the most frequently affected. Extremes of age were seen in 15 (57.7%) cases; 8 patients were elderly and 7 were younger than 1 year, of which only 2 were premature infant. In 15 cases, additional methods showed that the microorganisms isolated from biological samples were compatible with the probiotics taken by the patients. Antimicrobial susceptibility profile was not available for 46.1% of the cases. Most of the isolated microorganisms had proven sensitivity to penicillins and cephalosporins (Additional file 1: Table S7). Death occurred in only 1 (3.8%) case.

#### *Bifidobacterium spp*.

Of the 12 patients with infectious complications due to *Bifidobacterium spp.*, 10 were newborns (9 preterm). Enteral feeding was present in 10 cases and central venous access in 9 of them. *B. longum* was present in 6 cases of infectious complications, followed by *B. infantis* and *B. breve*, with two cases each. In one case, two species were isolated in blood cultures (*B. infantis* and *B. longum),* and in another case, there was no identification at the species level. In only 5 cases the sensitivity profile was given and all were sensitive to penicillins and cephalosporins (Additional file 1: Table S7). There were no deaths in this group.

#### *Bacillus spp*.

One case series described 4 cases of bacteremia caused by *B. subtilis* associated to the use of probiotics containing these microorganisms; the probiotic was given to reduce the number of diarrheal episodes related to enteral nutrition. The antimicrobial sensitivity profile was identical between the microorganisms isolated in the blood and those found in the administered probiotics. Another case, reported a decade later, was of sepsis due to *B. subtilis* in in a 73-year-old man with chronic lymphocytic leukemia who used probiotics before his hospitalization. The same *B. subtilis* strain, identified by a deoxyribonucleic acid (DNA) amplification technique, was found in the patient’s blood. The antimicrobial sensitivity profile was performed in only 1 case and the organism was sensitive to penicillins, cephalosporins, aminoglycosides and glycopeptides (Additional file 1: Table S7). Three of the 5 affected patients died.

### Gender and age distribution, and associated conditions

Males had a slightly higher prevalence, corresponding to 53 (57%) of the cases. Elderly (defined as adults over 60 years of age) and children younger than 1 year were reported in 33 (35.5%) and 25 (26.7%) cases, respectively. Of patients younger than 1 year, 17 (68%) were premature (Additional file 1: Table S8). Central venous access, enteral nutrition and parenteral nutrition were present in 59 (63.5%), 52 (56%) and 14 (15%), respectively, of all cases. Neoplasia was described in 10 (10.7%) and of these, 4 (40%) were of hematologic malignancies. Patients on immunosuppressive drugs (corticosteroids, chemotherapy and immunobiologicals) accounted for 14 (15%) patients. Two patients had received solid organ transplantation and 5 (5.4%) had been previously diagnosed as HIV infected. At least 40 (43%) of the cases were on antimicrobial therapy at the time of the infectious complications and 11 (11.8%) had *C. difficile* colitis. Blood was the most frequent site of isolation of probiotic microorganisms (Table 2). All-cause mortality was 19.6% (18 cases) and it was 22.8% among the 57 cases where there was compatibility between probiotics and the isolated microorganisms in the biological samples.

**Table 2:** Biological samples from which microorganisms were isolated in 93 patients who used probiotics

Biological material	Absolute value (%)
Blood	87 (93.5)
Retropharyngeal abscess fluid	1 (1.1)
Liver abscess fluid	2 (2.1)
Pleural fluid	1 (1.1)
Synovial fluid	1 (1.1)
Broncheoalveolar lavage fluid	1 (1.1)
Total	93 (100)

### Factors associated with mortality

The mean age was higher in the group that died, as were the prevalence of *C. difficile* colitis and antibiotic use during the infectious complication. The group of patients with infectious complications by *Saccharomyces spp.* had a higher death rate (Table 3).

**Table 3:** Univariate analysis of clinical and microbiological predictors of death in 93 cases of infectious complications after probiotic use, identified by systematic review, 1976–2018

	No-death (*n* = 74)	Death (*n* = 18)	*p*-value	OR (95% CI)
Male, n (%)	45 (60.8)	8 (44.4)	0.43	–
Age (years)	33.8 ± 31^a^	59.4 ± 22^a^	< 0.001^*^	–
Preterm, n (%)	16 (21.6)	1 (5.5)	0.03^*^	0.10 (0.01–1.91)
> 60 years, n (%)	22 (29.7)	11 (61.1)	0.003^*^	6.25 (1.89–20.1)
CDc, n (%)	4 (5.4)	7 (38.9)	< 0.001^*^	11.8 (2.89–48.81)
HIV infection	5 (6.7)	0	0.23	–
Solid organ transplant	2 (2.7)	1 (5.5)	0.55	–
Immunosuppressive drugs	15 (20.2)	5 (27.8)	0.89	–
Enteral nutrition	38 (51.3)	13 (72.2)	0.13	–
Parenteral nutrition	12 (16.2)	2 (11.1)	0.37	–
Central venous catheter	48 (64.9)	11 (61.1)	0.96	–
ATB use, n (%)	28 (37.8)	12 (66.7)	0.002^*^	9.75 (1.51–17.11)
Etiology				
*Saccharomyces spp.*	32 (43.2)	14 (77.8)	0.03^*^	4.9 (1.30–15.48)
*Lactobacillus spp.*	25 (33.8)	1 (5.5)	0.03^*^	0,12 (0.01–1.05)
*Bifidobacterium spp.*	12 (16.2)	0 (0)	0.11	0,12 (0.01–2.31)
Beginning of probiotics until symptoms	11 (7–23)^b^	9 (8–14)^b^	0,30	–
Duration of treatment (d)	20 ± 16^a^	11 ± 8^a^	0.20	–

*ATB* Antibiotics, *CDc* C. difficile colitis, *(d)* Days, *CI* 95% confidence interval, *OD* Odds ratio

*p-value statistically significant

^a^Median and interquartile

^b^mean and standard deviation

## Discussion

This systematic review of original articles published between 1976 and 2018 brought together 93 cases of patients who developed infectious complications related to probiotic ingestion.

The geographical distribution of cases identified by this review covers the 5 continents, and included developed as well as developing countries. We believe that these cases represent only a small proportion of existing cases, since case reports are increasingly difficult to publish. Not only the geographical dispersion of cases but also the increasing number of cases over time are noteworthy, possibly due to the more intensive use of probiotics around the world.

The most frequent infectious complication was fungemia, with 37.6% of the cases. *Saccharomyces* were the most frequently reported probiotic microorganisms, accounting for nearly half of the cases. When evaluating sepsis only, 39.3% were caused by these fungi. The incidence of fungemia by *S. cerevisiae* is unknown, although population studies associate it with 0.1 to 3.6% of all episodes of fungemia [15]. Currently, *S. boulardii*, which is marketed in many countries as a medicine to treat gastroenteritis, is considered identical to a *S. cerevisiae* strain; this fact corroborates the findings of some authors about the compatibility between *S. cerevisiae* identified in the biological material of the patients and *S. boulardii* isolated from the probiotics of the respective cases. A review of fungemia [15] by *S. cerevisiae* showed that of the 60 cases found in the literature, 26 (43%) had previously used probiotics. Treatment of *Saccharomyces* infections is based on expert opinion, and most clinical experience exists with fluconazole and amphotericin B; besides systemic antifungals, it is strongly recommended that probiotics are discontinued and indwelling foreign bodies are removed [16]. In our review, 12 samples of *Saccharomyces spp.* had antimicrobial susceptibility testing and 10 of them were sensitive to azoles. It is interesting to note that in the systematic review on use of probiotics in critical illness, only the older studies (from the 80’s and 90’s) had *Saccharomyces* as the prescribed probiotic [2].

Bacteremia accounted 20.4% of the infectious complications and the genus *Lactobacillus* was responsible for 42% of these. *Lactobacillus* bacteremia has an annual incidence of approximately 0.1 to 0.3%, [7] in Finland. The 26% mortality at 30 days described in a recent review [17] was credited to patients’ underlying disease, rather than bacteremia alone. The risk factors described for the occurrence of lactobacillemia are the use of broad-spectrum antimicrobials, invasive procedures of the gastrointestinal or respiratory tract, immunosuppressive conditions and selective intestinal decontamination [18, 19]. We observed that of the 8 patients with *Lactobacillus* bacteremia, 4 were immunosuppressed - 2 with HIV infection; 3 underwent intestinal invasive procedures and 1 had diverticular disease of the colon. There are some clinical studies with the use of probiotics in HIV patients where no infectious adverse effects have been reported. The risk of infection by probiotics in HIV patients appears to be small, but attention should be paid to patients with low CD4 count and disease or manipulation of the intestinal tract [20, 21].

Boyle et al. [22], evaluating children, proposed some probiotic risk factors for sepsis, and impaired immune system and preterm birth were considered the most important risk factors. In our review, 26.7% of the patients were younger than 1 year of age and practically two-thirds of them premature. Ten preterm infants had extremely low birth weight and half had sepsis, four patients had bacteremia and only one had fungemia. In 10 patients the indication for the use of probiotic was to prevent necrotizing enterocolitis (NE) and at least 4 of them ended up developing this complication, despite probiotic prophylaxis. NE is a serious condition, characterized by breakage of the intestinal barrier, dysbiosis and persistent inflammation of the colon; surgery may be necessary in 20 to 40% of cases and mortality may reach 30% [23]. The systematic review and meta-analysis of Sawh et al. [24], which evaluated the efficacy and safety of probiotics in more than 5000 infants less than 37 weeks of gestation or weighing less than 2500 g, observed a reduction in the incidence of severe NE and overall mortality compared to the placebo group. However, in the extremely low birthweight group, no significant difference was demonstrated for these outcomes. In a recently published large (4556 subjects), double-blind, placebo-controlled trial of an oral synbiotic preparation (*Lactobacillus plantarum* plus fructooligosaccharide) in rural Indian newborns, a significant reduction in the primary outcome (combination of sepsis and death) was found in the treatment arm (risk ratio 0.60, 95% confidence interval 0.48–0.74). Also significant reductions were also observed for culture-positive and culture-negative sepsis and lower respiratory tract infections. The authors suggest that a large proportion of neonatal sepsis in developing countries may be avoided through this strategy [25], although we believe safe drinking water and sanitation are more important for global health.

In our study, all-cause mortality was associated with age over 60 years, *C. difficile* colitis, and antibiotic use at the time of the probiotic-related infectious complication. The genus *Saccharomyces* was also positively associated with mortality, since 3/4 of patients who died had probiotics belonging to this genus as the etiological agent of the infectious complications found. Although the analysis suggests that the genus *Saccharomyces* is associated with higher overall mortality, we know that other factors were not considered, such as the numbers and types of comorbidity.

This systematic review is not intended to discourage the use of probiotics, which have been shown to be effective in many situations in clinical practice. Nonetheless, most trials evaluating probiotics included patients that were not severely ill, and therefore serious adverse events were not expected, which is often not a real-life scenario. On the other hand, the largest to date systematic review on the use of probiotics in critically ill patients failed to demonstrate an effect on ICU or hospital mortality. The number of patients included per trial was small, and the variety of probiotic strains, wide range of daily doses, and length of administration among the different trials weakened any possible clinical conclusions and recommendations [2] . Therefore, to assume that probiotic intake is completely risk-free is not true. The proportion of cases of infectious complications is small when the total number of people who use probiotics is considered. However, the cases described here are infections with high mortality rates such as endocarditis and sepsis. So, although on one hand there is the possibility of publication bias, with more serious cases having been published, on the other, due to the mentioned limitation for the publication of case reports, several other serious cases may not have reached public knowledge.

## Conclusion

The most frequent probiotic-related infectious complications were fungemia and sepsis and the most frequent probiotic microorganisms were of the genus *Saccharomyces*, a fungus.

Mortality was associated with age >  60 years, *C. difficile* colitis, current antimicrobial use and *Saccharomyces* infection.

Probiotics were often used in the context of excessive antibiotic use, and a more judicious use of antibiotics is critical, as the use of probiotics cannot be considered risk free and should be carefully evaluated for high- risk groups of patients.

## Additional file

Additional file 1: **Supplement 1.** Characteristics of searches in bibliographic databases. **Supplement 2.** Form for extracting data from case reports. **Supplement 3.** Quality Assessment Tool for Case Series Studies. **Supplement 4.** Items excluded in the evaluation of articles for eligibility. **Supplement 5.** Case reports included for qualitative synthesis. **Table S1.** Cases of fungemia after use of probiotics in 35 patients identified by systematic review, 1976–2018. Clinical details of each case of fungemia. **Table S2.** Sepsis cases after use of probiotics in 29 patients identified by systematic review, 1976–2018. Clinical details of each case of sepsis. **Table S3.** Cases of bacteremia after use of probiotics in 19 patients identified by systematic review, 1976–2018. Clinical details of each case of bacteremia. **Table S4.** Cases of endocarditis after using probiotics in 4 patients identified by systematic review, 1976–2018. Clinical details of each case of endocarditis. **Table S5.** Cases of abscess, empyema, septic arthritis and pneumonia after use of probiotics in 6 patients identified by systematic review, 1976–2018. Clinical details of each case of abscess, empyema and others complications. **Table S6.** Antimicrobial susceptibility test standard of Saccharomyces spp. of patients with infectious complication following probiotic use, identified by systematic review, 1976–2018. Antimicrobial susceptibility profile of *Saccharomyces spp*. **Table S7.** Antimicrobial susceptibility test standard for isolates of probiotic-related microorganisms from patients with infectious complications following probiotic use, identified by systematic review, 1976–2018. Antimicrobial susceptibility profile of *Lactobacillus spp*, *Bifidobacterium spp*, *Bacillus spp* and *Pediococcus spp*. **Table S8.** Patients younger than 1 year with infectious complication following probiotic use, identified by systematic review, 1976–2018. Clinical details of patients younger than 1 year. (DOCX 98 kb)

## Abbreviations

AIDS: Acquired immunodeficiency syndrome
DNA: Deoxyribonucleic acid
HIV: Human immunodeficiency virus
NE: Necrotizing enterocolitis
PRISMA: Preferred reporting items for systematic reviews and meta-analyzes
