# Supplementary material for: Infectious complications following probiotic ingestion: a potentially underestimated problem? A systematic review of reports and case series
Source: BMC Complement Altern Med. 2018 Dec 12;18:329. doi: 10.1186/s12906-018-2394-3 (PMC6292120; doi:10.1186/s12906-018-2394-3)
Supplement: Supplementary file 1 — Supplement 1. Characteristics of searches in bibliographic databases. Supplement 2. Form for extracting data from case reports. Supplement 3. Quality Assessment Tool for Case Series Studies. Supplement 4. Items excluded in the evaluation of articles for eligibility. Supplement 5. Case reports included for qualitative synthesis. Table S1. Cases of fungemia after use of probiotics in 35 patients identified by systematic review, 1976–2018. Clinical details of each case of fungemia. Table S2. Sepsis cases after use of probiotics in 29 patients identified by systematic review, 1976–2018. Clinical details of each case of sepsis. Table S3. Cases of bacteremia after use of probiotics in 19 patients identified by systematic review, 1976–2018. Clinical details of each case of bacteremia. Table S4. Cases of endocarditis after using probiotics in 4 patients identified by systematic review, 1976–2018. Clinical details of each case of endocarditis. Table S5. Cases of abscess, empyema, septic arthritis and pneumonia after use of probiotics in 6 patients identified by systematic review, 1976–2018. Clinical details of each case of abscess, empyema and others complications. Table S6. Antimicrobial susceptibility test standard of Saccharomyces spp. of patients with infectious complication following probiotic use, identified by systematic review, 1976–2018. Antimicrobial susceptibility profile of Saccharomyces spp. Table S7. Antimicrobial susceptibility test standard for isolates of probiotic-related microorganisms from patients with infectious complications following probiotic use, identified by systematic review, 1976–2018. Antimicrobial susceptibility profile of Lactobacillus spp, Bifidobacterium spp, Bacillus spp and Pediococcus spp. Table S8. Patients younger than 1 year with infectious complication following probiotic use, identified by systematic review, 1976–2018. Clinical details of patients younger than 1 year. (DOCX 98 kb) [file 12906_2018_2394_MOESM1_ESM.docx]

**Appendices - Supplementary material**

**Supplement 1 – Characteristics of searches in bibliographic databases**

Database: PubMed

Address: www.pubmed.org

Search Date: 08/07/2018

Period covered by the search: 1946-2018

There were no language restrictions

| **Search** | **Strategy** | **Result** |
| --- | --- | --- |
| #3 | #1 AND #2 | 1.747 |
| #2 | "sepsis" [mesh] OR "bacteremia" [mesh] OR "fungemia" [mesh] OR "endocarditis" [mesh] OR "abscess" [mesh] OR "infection" [mesh] | 756.113 |
| #1 | "probiotics" [mesh] OR "lactobacillus" [mesh] OR "bifidobacterium" [mesh] OR "saccharomyces" [mesh] OR "Yogurt" [mesh] | 141.259 |

Database: SciELO

Address: www.scielo.org

Search Date: 08/08/2018

Period covered by the search: 1998-2018

There were no language restrictions

| **Search** | **Strategy** | **Result** |
| --- | --- | --- |
| 1 | Probiotics [All index] AND sepsis [All index] | 8 |
| 2 | Probiotics [All index] AND bacteremia [All index] | 2 |
| 3 | Probiotics [All index] AND fungemia [All index] | 1 |
| 4 | Probiotics [All index] AND endocarditis [All index] | 0 |
| 5 | Probiotics [All index] AND abscess [All index] | 0 |
| 6 | Probiotics [All index] AND infection [All index] | 45 |
| 7 | Lactobacillus [All index] AND sepsis [All index] | 0 |
| 8 | Lactobacillus [All index] AND bacteremia [All index] | 0 |
| 9 | Lactobacillus [All index] AND fungemia [All index] | 0 |
| 10 | Lactobacillus [All index] AND endocarditis [All index] | 0 |
| 11 | Lactobacillus [All index] AND abscess [All index] | 0 |
| 12 | Lactobacillus [All index] AND infection [All index] | 42 |
| 13 | Bifidobacterium [All index] AND sepsis [All index] | 2 |
| 14 | Bifidobacterium [All index] AND bacteremia [All index] | 0 |
| 15 | Bifidobacterium [All index] AND fungemia [All index] | 0 |
| 16 | Bifidobacterium [All index] AND endocarditis [All index] | 0 |
| 17 | Bifidobacterium [All index] AND abscess [All index] | 0 |
| 18 | Bifidobacterium [All index] AND infection [All index] | 8 |
| 19 | Saccharomyces [All index] AND sepsis [All index] | 3 |
| 20 | Saccharomyces [All index] AND bacteremia [All index] | 0 |
| 21 | Saccharomyces [All index] AND fungemia [All index] | 3 |
| 22 | Saccharomyces [All index] AND endocarditis [All index] | 1 |
| 23 | Saccharomyces [All index] AND abscess [All index] | 0 |
| 24 | Saccharomyces [All index] AND infection [All index] | 8 |
| 25 | Yogurt [All index] AND sepsis [All index] | 0 |
| 26 | Yogurt [All index] AND bacteremia [All index] | 0 |
| 27 | Yogurt [All index] AND fungemia [All index] | 0 |
| 28 | Yogurt [All index] AND endocarditis [All index] | 0 |
| 29 | Yogurt [All index] AND abscess [All index] | 0 |
| 30 | Yogurt [All index] AND infection [All index] | 4 |

Database: Scopus

Address: www.scopus.com

Search Date: 08/06/2018

Period covered by the search: 1986-2018

There were no language restrictions

| **Search** | **Strategy** | **Result** |
| --- | --- | --- |
| 1 | Probiotics AND infection | 4923 |

**Supplement 2 – Form for extracting data from case reports**

Infectious complications following probiotic ingestion: systematic review of reports and case series.

Reviewer:_______ Article code:_____ Date:__/__/__ Seem: [ ] Approves [ ] Disapproved

**1. Publication data:**

1.1 Article title: __________________________________________________________________________________________________________________________________________________________________

1.2 Authors: __________________________________________________________________________________________________________________________________________________________________

1.3 Reference

1.3.1 Name of the journal_____________________________________________________________

1.3.2 Journal Year:________ 1.3.3 Volume and journal number: _____/______

1.3.4 Home page and end of the article:____/____

1.3.5 Contact Author:__________________________________________________________

**2. Country of publication:**____ **3. Age of patient:**___ **4. Gender: [ ] M [ ]F**

**5. Morbidity:** [ ] Preterm [ ] HIV/AIDS [ ] Elderly [ ] Solid Organ Transplantation

[ ] Immunosuppressed [ ] Others __________________________________________________________________________________________________________________________________________________________________

**6. Microorganism involved:**________________________________________________________

**7. Antimicrobial sensitivity profile:** Sensitivity ________________________________

_________________________________________________________________________________

Resistance: ______________________________________________________________________

_________________________________________________________________________________

**8. Biological material of the microorganism identification**:________________________________

**9. Type of infection: _______________________________________________________________**

**10. Duration of treatment: _________________________________________________________**

**11. Death:** [ ] Yes [ ] No

**12. Comments: __________________________________________________________________ _______________________________________________________________________________________________________________________________________________________________**

**Supplement 3 – Quality Assessment Tool for Case Series Studies**

| Criteria | Yes | No | NA, NR |
| --- | --- | --- | --- |
| Was the study question or objective clearly stated? |  |  |  |
| Was the study population clearly and fully described, including a case definition? |  |  |  |
| Were the cases consecutive? |  |  |  |
| Were the subjects comparable? |  |  |  |
| Was the intervention clearly described? |  |  |  |
| Were the outcome measures clearly defined, valid, reliable, and implemented consistently across all study participants? |  |  |  |
| Was the length of follow-up adequate? |  |  |  |
| Were the statistical methods well-described? |  |  |  |
| Were the results well-described? |  |  |  |

NA, not applicable; NR, not reported

NIH/National Institutes of Health. Disponível em: https://www.nhlbi.nih.gov/health-pro/guidelines/in-develop/cardiovascular-risk-reduction/tools/case_series Access: 10/21/2016

**Supplement 4 – Items excluded in the evaluation of articles for eligibility.**

| **Article title** | **Reason for exclusion** |
| --- | --- |
| 65-year-old woman with confusion. | No previous use of probiotic |
| A case of an atypical femoral fracture associated with bacterial biofilm--pathogen or bystander? | No previous use of probiotic |
| A case of *Lactobacillus acidophilus* endocarditis successfully treated with cefazolin and gentamicin. | No previous use of probiotic |
| A case of *Lactobacillus casei* bacteraemia associated with aortic dissection: is there a link? | No previous use of probiotic |
| A curious case of *Lactobacillus casei* in a prosthetic joint: was it the yogurt? | Possible contamination by vaginal flora |
| A rare case of *Lactobacillus acidophilus* presenting as mitral valve bacterial endocarditis. | No previous use of probiotic |
| A yeast-like infection of the esophagus caused by *Lactobacillus acidophilus*. | No previous use of probiotic |
| Abscess caused by vancomycin resistant *Lactobacillus confuses.* | No previous use of probiotic |
| Acute acalculous cholecystitis complicated with peritonitis caused by *Lactobacillus plantarum.* | No previous use of probiotic |
| Acute infectious pseudoaneurysm of the descending thoracic aorta and review of infectious aortitis. | No previous use of probiotic |
| An unusual case of splenic abscess and sepsis in an immunocompromised host. | No previous use of probiotic |
| Aortic graft infection by *Lactobacillus casei*: a case report. | No previous use of probiotic |
| Apropos of a case of endocarditis due to a *Lactobacillus*. | No previous use of probiotic |
| Are live *Saccharomyces* yeasts harmful to patients? | Does not provide case data |
| Association of *Lactobacillus plantarum* with endocarditis. | No previous use of probiotic |
| Bacteremia and pyelonephritis caused by *Lactobacillus jensenii* in a patient with urolithiasis. | No previous use of probiotic |
| Bacteremia caused by *Lactobacillus plantarum* in endocarditis lenta. | No previous use of probiotic |
| Bacteremia due to *Bifidobacterium*, *Eubacterium* or *Lactobacillus*; twenty-one cases and review of the literature. | No previous use of probiotic |
| Bacterial associated porcine heterograft heart valve calcification. | No previous use of probiotic |
| *Bifidobacterium* (*Actinomyces*) *eriksonii* Infection. | No previous use of probiotic |
| *Bifidobacterium* species bacteremia: risk factors in adults and infants. | No previous use of probiotic |
| *Bifidobacterium*--friend or foe? A case of urinary tract infection with *Bifidobacterium* species. | No previous use of probiotic |
| Bilateral ureteral obstruction due to *Saccharomyces cerevisiae* fungus balls. | No previous use of probiotic |
| CAPD peritonitis caused by vancomycin-resistant *lactobacilli.* | No previous use of probiotic |
| CAPD peritonitis caused by *Lactobacillus rhamnosus.* | No previous use of probiotic |
| Case of aortic endocarditis caused by *Lactobacillus casei.* | No previous use of probiotic |
| Case of sepsis caused by *Bifidobacterium longum.* | No previous use of probiotic |
| Case report: fatal anaerobic pulmonary infection due to *Bifidobacterium eriksonii*. | No previous use of probiotic |
| Catheter-related bacteremia due to *Lactobacillus rhamnosus* in a single-lung transplant recipient. | No previous use of probiotic |
| Catheter-related fungemia caused by *Saccharomyces cerevisiae* in a newborn. | No previous use of probiotic |
| Cervical epidural abscess and vertebral osteomyelitis following non-traumatic oesophageal rupture: a case report and discussion. | No previous use of probiotic |
| Chest infection caused by *Lactobacillus casei ss rhamnosus.* | No previous use of probiotic |
| Chondritis attributable to *Lactobacillus* after ear piercing. | No previous use of probiotic |
| Chorioamnionitis and possible neonatal infection associated with *Lactobacillus species*. | No previous use of probiotic |
| Clinical manifestations and therapy of *Lactobacillus endocarditis*: report of a case and review of the literature. | No previous use of probiotic |
| Clones of *Lactobacillus casei* and *Torulopsis glabrata* associated with recurrent abdominal wall abscess. | No previous use of probiotic |
| Deep seated infection due to *Lactobacillus caseii.* | No previous use of probiotic |
| Descending necrotizing mediastinitis associated with *Lactobacillus plantarum*. | No previous use of probiotic |
| Disseminated bread yeast fungaemia in a baker's wife with acute myeloid leukaemia. | No previous use of probiotic |
| Disseminated infection with *Saccharomyces kluyveri* in a patient with AIDS. | No previous use of probiotic |
| Disseminated *Saccharomyces cerevisiae* infection following polymicrobial hepatobiliary sepsis. | No previous use of probiotic |
| Does there exist a chronic bacillary septicemia? A clinical case report. | No previous use of probiotic |
| Does yoghurt gnaw at cardiac valves? | Article in German |
| Emerging opportunistic yeast infection. | No previous use of probiotic |
| Endocarditis caused by *Lactobacillus casei suspecies rhamnosus*. | No previous use of probiotic |
| Endocarditis due to rare and fastidious bacteria. | Review article |
| Endocarditis verrucosa secundaria a *Saccharomyces cerevisiae*: clinical case. | No previous use of probiotic |
| Endocarditis caused by *diphtheroids* and *lactobacilli.* | No previous use of probiotic |
| Endocarditis caused by Lactobacillus. | No previous use of probiotic |
| Endocarditis caused by *Lactobacillus casei subspecies rhamnosus*. A case report. | No previous use of probiotic |
| Endocarditis caused by *Lactobacillus casei*. Apropos of a case. | Unavailable |
| Endocarditis caused by *Lactobacillus jensenii* in an immunocompetent patient. | No previous use of probiotic |
| Endocarditis caused by *Lactobacillus plantarum.* | No previous use of probiotic |
| Endocarditis caused by*Lactobacillus*. | No previous use of probiotic |
| Endocarditis caused by *Saccharomyces cerevisiae* on the prosthetic valve. | No previous use of probiotic |
| Endocarditis due to *Lactobacillus acidophilus* in a patient with structural heart disease. | No previous use of probiotic |
| Endocarditis due to *Lactobacillus casei/paracasei.* | No previous use of probiotic |
| Endocarditis due to *Lactobacillus plantarum*. Description of a case and review of the literature. | No previous use of probiotic |
| Endocarditis in older people. | No previous use of probiotic |
| Endocarditis lenta caused by *Lactobacillus salivarius subsp. Salicinicus.* | No previous use of probiotic |
| Endocarditis of the native aortic valve caused by *Lactobacillus jensenii.* | No previous use of probiotic |
| Epidural abscess due to *Streptococcus milleri* and *Bifidobacterium species.* | No previous use of probiotic |
| Fatal lung abscess due to *Lactobacillus casei ss rhamnosus.* | No previous use of probiotic |
| Food and probiotics trainsfrom the *Saccharomyces cerevisiae* species as a possible origin of human systemic infection. | No previous use of probiotic |
| Four hours for a record, or a severe fuminating cellulitis: can *Saccharomyces cerevisiae* be the causal agent? | No previous use of probiotic |
| Fungal endocarditis in critically ill children. | No previous use of probiotic |
| Fungal endocarditis: analysis of 24 cases and review of the literature | No previous use of probiotic |
| Fungal peritonitis in children on continuous ambulatory peritoneal dialysis. | No previous use of probiotic |
| Hepatic abscess and bacteremia due to *Lactobacillus rhamnosus.* | No previous use of probiotic |
| How safe is safe?--a case of *Lactobacillus paracasei ssp.* paracasei endocarditis and discussion of the safety of lactic acid bacteria. | No previous use of probiotic |
| Identification by 16S rRNA gene sequencing of *Lactobacillus salivarius* bacteremic cholecystitis | No previous use of probiotic |
| Identification of lactic acid bacteria isolated from human blood cultures. | No previous use of probiotic |
| Identification of *Lactobacillus* strains from patients with infective endocarditis and comparison of their surface associated properties with those of other strains of the same species. | Observational study |
| Infectious keratitis caused by *Stenotrophomonas maltophilia* and yeast simultaneously. | No previous use of probiotic |
| Infectious endocarditis caused by *Lactobacillus acidophilus* in a patient with mistreated dental caries. | No previous use of probiotic |
| Infective endocarditis in adults. | Review article |
| Infective fungal endocarditis. | No previous use of probiotic |
| Infective endocarditis caused by *lactobacillus*. | No previous use of probiotic |
| Infective endocarditis due to *Lactobacillus acidophilus* group. Report of a case and review of the literature. | No previous use of probiotic |
| Intravascular infection with *Lactobacillus paracasei.* | No previous use of probiotic |
| Invasive infection with *Saccharomyces cerevisiae*: report of three cases and review. | No previous use of probiotic |
| Invasive *Saccharomyces cerevisiae* in a liver transplant patient: case report and review of infection in transplant recipients. | No previous use of probiotic |
| Invasive *Saccharomyces cerevisiae* infection: a friend turning foe? | No previous use of probiotic |
| Isolated*Lactobacillus* chronic prosthetic knee infection. | No previous use of probiotic |
| Isolation of vancomycin-resistant lactobacilli from three neutropenic patients with pneumonia. | No previous use of probiotic |
| *Lactobacillemia* in pregnancy. | No previous use of probiotic |
| Lactobacillemia in three patients with AIDS | No previous use of probiotic |
| Lactobacillemia of renal origin: a case report. | No previous use of probiotic |
| Lactobacillemia--report of nine cases. Important clinical and therapeutic considerations. | No previous use of probiotic |
| *Lactobacilli* and pleuropulmonary infection. | No previous use of probiotic |
| *Lactobacilli* and urinary tract infection. | No previous use of probiotic |
| *Lactobacillus casei* endocarditis in tricuspid atresia. | Article in Arabic |
| *Lactobacillus* endocarditis: case report and literature review. | No previous use of probiotic |
| *Lactobacillus rhamnosus GG* suspected infection in a newborn with intrauterine growth restriction. | Unavaliable |
| *Lactobacillus rhamnosus* infection in a child following bone marrow transplant. | No previous use of probiotic |
| *Lactobacillus* species as a cause of ventilator-associated pneumonia in a critically ill trauma patient | No previous use of probiotic |
| *Lactobacillus acidophilus* as a cause of liver abscess in a NOD2/CARD15-positive patient with Crohn's disease. | No previous use of probiotic |
| *Lactobacillus acidophilus* peritonitis in CAPD. | No previous use of probiotic |
| *Lactobacillus acidophilus* endocarditis. | No previous use of probiotic |
| *Lactobacillus acidophilus* endocarditis after an appendectomy. | No previous use of probiotic |
| *Lactobacillus acidophilus* sepsis in a neonate. | No previous use of probiotic |
| *Lactobacillus* allograft pyelonephritis and bacteremia. | No previous use of probiotic |
| *Lactobacillus* and tuberculous empyema. | No previous use of probiotic |
| *Lactobacillus* bacteremia and endocarditis: review of 45 cases. | No previous use of probiotic |
| *Lactobacillus bacteremia*: description of the clinical course in adult patients without endocarditis. | No previous use of probiotic |
| *Lactobacillus casei* pneumonia and sepsis in a patient with AIDS. Case report and review of the literature. | Sem uso prévio de probiótico |
| *Lactobacillus casei subsp. rhamnosus* sepsis in a patient with ulcerative colitis. | No previous use of probiotic |
| *Lactobacillus casei subspecies casei* endocarditis--a case report. | No previous use of probiotic |
| *Lactobacillus casei* endocarditis. | No previous use of probiotic |
| *Lactobacillus casei* endocarditis after aortic valve prosthesis. | No previous use of probiotic |
| *Lactobacillus casei* endocarditis in an intravenous heroin drug addict: a case report. | No previous use of probiotic |
| *Lactobacillus casei* infection in an AIDS patient. | No previous use of probiotic |
| *Lactobacillus delbrueckii* as the cause of urinary tract infection. | No previous use of probiotic |
| *Lactobacillus delbrueckii*: probable agent of urinary tract infections in very old women. | No previous use of probiotic |
| *Lactobacillus* endocarditis. | No previous use of probiotic |
| *Lactobacillus* endocarditis. | No previous use of probiotic |
| *Lactobacillus* endocarditis. | No previous use of probiotic |
| *Lactobacillus* endocarditis: a case report of outpatient management. | No previous use of probiotic |
| *Lactobacillus* endocarditis: case report and review of cases reported since 1992. | No previous use of probiotic |
| *Lactobacillus fermentum* endocarditis involving a native mitral valve. | No previous use of probiotic |
| *Lactobacillus gasseri* as the cause of septic urinary infection. | No previous use of probiotic |
| *Lactobacillus* infective endocarditis. | No previous use of probiotic |
| *Lactobacillus* isolated pulmonic valve endocarditis with ventricular septal defect detected by transesophageal echocardiography. | No previous use of probiotic |
| *Lactobacillus jensenii* prosthetic valve endocarditis. | No previous use of probiotic |
| *Lactobacillus jensenii* bacteremia and endocarditis after dilatation and curettage: case report and literature review. | No previous use of probiotic |
| *Lactobacillus paracasei* continuous ambulatory peritoneal dialysis-related peritonitis and review of the literature. | No previous use of probiotic |
| *Lactobacillus paracasei* endocarditis in an 18-yeard-old patient with trisomy 21, atrioventricular septal defect and Eisenmenger complex: therapeutic problems. | No previous use of probiotic |
| *Lactobacillus peritonitis*: a rare cause of peritonitis in peritoneal dialysis patients. | No previous use of probiotic |
| *Lactobacillus plantarum* endocarditis. | No previous use of probiotic |
| *Lactobacillus plantarum* endocarditis in a patient with benign monoclonal gammopathy. | No previous use of probiotic |
| *Lactobacillus rhamnosus* hepatic abscess associated with Mirizzi syndrome: a case report and review of the literature. | No previous use of probiotic |
| *Lactobacillus rhamnosus* meningitis following recurrent episodes of bacteremia in a child undergoing allogeneic hematopoietic stem cell transplantation. | No previous use of probiotic |
| *Lactobacillus rhamnosus* septicaemia in a patient with a graft in the inferior vena cava. | No previous use of probiotic |
| *Lactobacillus rhamnosus* septicemia in patients with prolonged aplasia receiving ceftazidime-vancomycin. | No previous use of probiotic |
| *Lactobacillus rhamnosus* bacteremia in a kidney transplant recipiente. | No previous use of probiotic |
| *Lactobacillus rhamnosus* endocarditis complicating colonoscopy. | No previous use of probiotic |
| *Lactobacillus* sepsis with pelvic abscess. | No previous use of probiotic |
| *Lactobacillus* septic arthritis. | No previous use of probiotic |
| *Lactobacillus* septicemia, an unusual complication during the treatment of metastatic choriocarcinoma. | No previous use of probiotic |
| *Lactobacillus* species as emerging pathogens in neutropenic patients. | No previous use of probiotic |
| *Lactobacillus* species as opportunistic pathogens in immunocompromised patients. | No previous use of probiotic |
| *Lactobacillus spp.* bacteremia in a patient with neutropenia secondary to the treatment of acute leucemia. | No previous use of probiotic |
| *Leukocytoclastic angiitis* in subacute bacterial endocarditis. | No previous use of probiotic |
| Liver abscess caused by *Lactobacillus acidophilus.* | No previous use of probiotic |
| Liver abscess complicating intratumoral ethanol injection therapy for HCC. | No previous use of probiotic |
| Lung abscess and pleuritis caused by *Lactobacillus rhamnosus* in an immunocompetent patient. | No previous use of probiotic |
| Managing embolic myocardial infarction in infective endocarditis: current options. | No previous use of probiotic |
| Molecular diagnosis of endocarditis due to *Lactobacillus casei* *subsp. Rhamnosus.* | No previous use of probiotic |
| Native-valve endocarditis produced by *Lactobacillus casei* *sub. rhamnosus* refractory to antimicrobial therapy. | No previous use of probiotic |
| Neonatal meningitis due to *Lactobacillus*. | No previous use of probiotic |
| New spectrum of fungal infections in patients with cancer. | No previous use of probiotic |
| Nosocomial transmission of *Saccharomyces cerevisiae* in bone marrow transplant patients. | No previous use of probiotic |
| Observation of *Saccharomyces cerevisiae* in blood of patient undergoing root canal treatment. | No previous use of probiotic |
| Opportunistic osteomyelitis in the jaws of children on immunosuppressive chemotherapy. | No previous use of probiotic |
| Outbreak of *Saccharomyces cerevisiae* subtype boulardi fungemia in patients neighboring those treated with a probiotic preparation of the organism. | No previous use of probiotic |
| Peritonitis associated with vancomycin-resistant *Lactobacillus rhamnosus* in a continuous ambulatory peritoneal dialysis patient: organism identification, antibiotic therapy, and case report. | No previous use of probiotic |
| Peritonitis caused by *Bifidobacterium longum*: case report and literature review. | No previous use of probiotic |
| Peritonitis caused by *Saccharomyces cerevisiae* in an ambulatory peritoneal dialysis patient. | No previous use of probiotic |
| Peritonitis due to *Saccharomyces cerevisiae* in a patient on CAPD. | No previous use of probiotic |
| Persistent *Lactobacillus casei subspecies rhamnosus* bacteremia in a 14 year old girl with acute myeloid leukemia. A case report. | No previous use of probiotic |
| Post-ERCP bacteriemia due to *Lactobacillus casei*: a case history. | No previous use of probiotic |
| Postoperative peritonitis caused by *Saccharomyces cerevisiae.* | No previous use of probiotic |
| Postoperative peritonitis due to *Saccharomyces cerevisiae* --treated with ketoconazole. | No previous use of probiotic |
| Postpartum *Lactobacillus jensenii* endocarditis in patient with bicuspid aortic valve. | No previous use of probiotic |
| Posttraumatic endophthalmitis caused by *Lactobacillus.* | No previous use of probiotic |
| Primary peritonitis due to *Lactobacillus fermentum.* | No previous use of probiotic |
| Profound mycoses in AIDS in Abidjan (Côte d'Ivoire). | No previous use of probiotic |
| Pseudo-outbreak of toxigenic *Bacillus cereus* isolated from stools of three patients with diarrhoea after oral administration of a probiotic medication. | No previous use of probiotic |
| Purpura fulminans associated with *Lactobacillus paracasei* liver abscess. | No previous use of probiotic |
| Pyelonephritis and septicemia due to grampositive rods similar to *Corynebacterium* group E (aerotolerant *Bifidobacterium adolescentis*). | No previous use of probiotic |
| Recurrent infective endocarditis in idiopathic hypertrophic subaortic stenosis. | No previous use of probiotic |
| Recurrent urinary infection with *Bifidobacterium scardovii.* | No previous use of probiotic |
| Recurrent *Saccharomyces cerevisiae* fungemia in an otherwise healthy patient. | No previous use of probiotic |
| Rhinocerebral *Mucor circinelloides* infection in immunocompromised patient following yogurt ingestion. | Contaminated yogurt |
| *Saccharomyces cerevisiae* fungemia in a multiply traumatized patient. | No previous use of probiotic |
| *Saccharomyces cerevisiae* fungemia in an immunocompromised patient not treated with *Saccharomyces boulardii* preparation. | No previous use of probiotic |
| *Saccharomyces cerevisiae* oesophagitis in a patient with oesophageal carcinoma. | No previous use of probiotic |
| *Saccharomyces cerevisiae* pneumonia in a patient with acquired immune deficiency syndrome. | No previous use of probiotic |
| *Saccharomyces boulardii* fungemia. Apropos of a case. | Unavaliable |
| *Saccharomyces cerevisiae* as a cause of oral thrush & diarrhoea in an HIV/ AIDS patient. | No previous use of probiotic |
| *Saccharomyces cerevisiae* emboli in an immunocompromised patient with relapsed acute myeloid leukaemia. | No previous use of probiotic |
| *Saccharomyces cerevisiae* empyema in a patient with esophago-pleural fistula complicating variceal sclerotherapy. | No previous use of probiotic |
| *Saccharomyces cerevisiae* infections in man. | No previous use of probiotic |
| *Saccharomyces cerevisiae* oesophagitis in an HIV-infected patient. | No previous use of probiotic |
| *Saccharomyces cerevisiae* peritonitis complicating CAPD. | No previous use of probiotic |
| *Saccharomyces cerevisiae* septicemia. | No previous use of probiotic |
| *Saccharomyces cerevisiae* septicemia in a patient with myelodysplastic syndrome. | No previous use of probiotic |
| *Saccharomyces cerevisiae* fungemia with granulomas in the bone marrow in a patient undergoing BMT. | No previous use of probiotic |
| *Saccharomyces cerevisiae* fungemia: case report and review of the literature. | No previous use of probiotic |
| *Saccharomyces cerevisiae* sepsis in a 35-week-old premature infant. A case report. | No previous use of probiotic |
| *Saccharomyces cerevisiae*-associated diarrhea in an immunocompetent patient with ulcerative colitis. | No previous use of probiotic |
| *Saccharomyces* fungemia. | No previous use of probiotic |
| *Saccharomyces fungemia* in a patient with AIDS. | No previous use of probiotic |
| Saccharomyces keratitis and endophthalmitis. | No previous use of probiotic |
| Safety of lactic acid bacteria and their occurrence in human clinical infection. | Observational study |
| Self-inflicted bacteraemia and fungaemia in Vietnamese migrants. | No previous use of probiotic |
| Septic arthritis due to *Saccharomyces* species in a patient with chronic rheumatoid arthritis. | No previous use of probiotic |
| Septic coronary embolism. | No previous use of probiotic |
| Septicaemia due to *Lactobacillus jensenii*: bacteriological diagnostic orientation. | No previous use of probiotic |
| Site and clinical significance of *Alloscardovia omnicolens* and *Bifidobacterium* species isolated in the clinical laboratory. | No previous use of probiotic |
| Six cases of *Lactobacillus* bacteraemia: identification of organisms and antibiotic susceptibility and therapy. | No previous use of probiotic |
| Soft tissue abscess caused by *Torulopsis glabrata* and *Lactobacillus.* | No previous use of probiotic |
| Splenic abscess and empyema due to *Lactobacillus* species in an immunocompetent host. | No previous use of probiotic |
| Splenic abscess caused by Lactobacillus paracasei. | No previous use of probiotic |
| Subacute and acute endocarditis due to *Pseudomonas cepacia* in heroin addicts. | No previous use of probiotic |
| Subacute bacterial endocarditis caused by *lactobacilli* in child. | No previous use of probiotic |
| Subacute bacterial endocarditis due to Lactobacillus. | No previous use of probiotic |
| Submental abscess by *Lactobacillus acidophilus/jensenii.* | No previous use of probiotic |
| Successful tigecycline lock therapy in a *Lactobacillus rhamnosus* catheter-related bloodstream infection. | No previous use of probiotic |
| Successful treatment of early infective endocarditis and mediastinitis in a heart transplant recipient. | No previous use of probiotic |
| Successful two-stage revision of *Lactobacillus infection* of a total knee arthroplasty and literature review. | No previous use of probiotic |
| Surgical and long-term antifungal therapy for fungal prosthetic valve endocarditis. | No previous use of probiotic |
| Surgical site abscess caused by *Lactobacillus fermentum* identified by 16S ribosomal RNA gene sequencing. | No previous use of probiotic |
| “Swiss cheese-like" brain due to *Lactobacillus rhamnosus*. | No previous use of probiotic |
| The spectrum of non-*Candida* fungal infections following bone marrow transplantation. | No previous use of probiotic |
| The trouble in tracing opportunistic pathogens: cholangitis due to *Bacillus* in a French hospital caused by a strain related to an Italian probiotic? | Observational study |
| Transmission of *Lactobacillus* pneumonia by a transplanted lung. | No previous use of probiotic |
| Two cases of *Lactobacillus rhamnosus* infection and pancreatitis. | No previous use of probiotic |
| Two cases of *Saccharomyces cerevisiae* Fungemia in Patients with Hematologic Malignancies. | Korean language |
| Two cases of endocarditis due to *Lactobacillus* species: antimicrobial susceptibility, review, and discussion of therapy. | No previous use of probiotic |
| Urinary tract infections caused by *Lactobacillus*. | No previous use of probiotic |
| Use of 16S rRNA gene sequencing to identify *Lactobacillus casei* in septicaemia secondary to a paraprosthetic enteric fistula. | No previous use of probiotic |
| Vancomycin resistance of clinical isolates of *lactobacilli.* | No previous use of probiotic |
| Verrucous endocarditis secondary to *Saccharomyces cerevisiae*. A case report. | No previous use of probiotic |
| *Weissella confusa* (basonym: *Lactobacillusconfusus*) bacteremia: a case report. | No previous use of probiotic |
| Probiotics and infective endocarditis in patients with hereditary hemorrhagic telangiectasia: a clinical case and a review of the literature. | Other Possible Causes |
| *Lactobacillus gasseri*endocarditis on the aortic valve bioprosthesis - a case report. | Polish language |
| *Lactobacillus rhamnosus*endocarditis*:* An unusual culprit in a patient with Barlow's disease. | No previous use of probiotic |
| Pediatric Sepsis Secondary to an Occult Dental Abscess: A Case Report. | No previous use of probiotic |
| Bacteremia induced by *Bifidobacterium breve* in a newborn with cloacal exstrophy. | No previous use of probiotic |
| Liver abscess and bacteremia caused by *lactobacillus*: role of probiotics? Case report and review of the literature. | Other Possible Causes |
| *Saccharomyces kluyveri* Fungemia in an Infant with Severe Combined Immunodeficiency. | No previous use of probiotic |

**Supplement 5 – Case reports included for qualitative synthesis**

1. Jensen DP, Smith DL. Fever of unknown origin secondary to Brewer’s yeast ingestion. Arch Intern Med. 1976;136(3):332-3.

2. Barton LL, Rider ED. Bacteremic infection with *Pediococcus*: vancomycin-resistant opportunist. Pediatrics. 2001;107(4):775-6.

3. Kunz AN, Noel JN, Fairchok MP. Two cases of *Lactobacillus* bacteremia during probiotic treatment of short gut syndrome. J Pediatr Gastroenterol Nutr. 2004;38(4):457-8.

4. Land MH, Rouster-Stevens K, Woods CR, Cannon ML, Cnota J, ShettyAK. *Lactobacillus* Sepsis Associated With Probiotic Therapy. Pediatrics. 2005;115(1):178-81.

5. LeDoux D, LaBombardi VJ, Karter D. *Lactobacillus acidophilus* bacteraemia after use of a probiotic in a patient with AIDS and Hodgkin’s disease. Int J STD AIDS. 2006;17(4):280-2.

6. Luong ML, Sareyyupoglu B, Nguyen MH et al. *Lactobacillus* probiotic use in cardiothoracic transplant recipients: a link to invasive *Lactobacillus* infection? Transpl Infect Dis 2010: 12: 561-4.

7. Mehta A, Rangarajan S, Borate U. A cautionary tale for probiotic use in hematopoietic SCT patients– Lactobacillus acidophilus sepsis in a patient with mantle cell lymphoma undergoing hematopoietic SCT. Bone Marrow Transplant. 2013; 48(3):461–2.

8. Vahabnezhad E, Mochon AB, Wozniak LJ, Ziring DA. *Lactobacillus* Bacteremia Associated With Probiotic Use in a Pediatric Patient With Ulcerative Colitis. J Clin Gastroenterol. 2013;47:437-9.

9. Bush LM, De Almeida KNF, Martin G, Perez MT. Probiotic-Associated *Bifidobacterium* Septic Prosthetic Joint Arthritis. Infect Dis Clin Pract. 2014;22:e39-41.

10. Doern CD, Nguyen ST, Afolabi F, Burnhamd CD. Probiotic-Associated Aspiration Pneumonia Due to *Lactobacillus rhamnosus*. J Clin Microbiol. 2014;52(8):3124-6.

11. Haghighat L, Crum-Cianflone NF. The potential risks of probiotics Among HIV-infected persons: Bacteraemia due to *Lactobacillus acidophilus* and review of the literature. Int J STD AIDS. 2015. doi:10.1177/0956462415590725.

12. Aroutcheva A, Auclair J, Frappier M et al. Importance of Molecular Methods to Determine Whether a Probiotic is the Source of *Lactobacillus* Bacteremia. Probiotics & Antimicro. Prot. 2016;8:31–40.

13. Viggiano M, Badetti C, Bernini V, Garabedian M, Janelli JC. *Saccharomyces boulardii* fungaemia in a severe burn patient. *Ann Fr Anesth Réanim.* 1995;14:356-8.

14. Boucaud C, Berrada K, Bouletraeau P. Septicémie à *Saccharomyces boulardii* après administration orale d'ultra-levure. Réanimation 1996;5(5):665.

15. Fredenucci I, Chomarat M, Boucaud C, Flandrois JP. *Saccharomyces boulardii* fungemia in a patient receiving Ultra-levure therapy. CID. 1998;27(1):222-3.

16. Niault M, Thomas F, Prost J, Ansari FH, Kalfon P. Fungemia Due to *Saccharomyces* Species in a Patient Treated with Enteral *Saccharomyces boulardii*. Clin Infect Dis. 1999;28(4):930.

17. Hennequin C, Kauffmann-Lacroix C, Jobert A et al. Possible Role of Catheters in *Saccharomyces boulardii* Fungemia. Eur J Clin Microbiol Infect Dis. 2000;19(1):16-20.

18. Lherm T, Monet C, Nougière B et al. Seven cases of fungemia with *Saccharomyces boulardii* in critically ill patients. Intensive Care Med. 2002;28(6):797-801.

19. Piechno S, Seguin P, Gangneux JP. Fongémie à *Saccharomyces boulardii*: méfiez-vous de la levure [Saccharomyces boulardii fungal sepsis: beware of the yeast]. Can J Anaesth. 2007;54(3):245-6.

20. Franko B, Vaillant M, Recule C, Vautrin E, Brion JP, Pavese P. *Lactobacillus paracasei* endocarditis in a consumer of probiotics. Med Mal Infect. 2013;43(4):171-3.

21. Oggioni MR, Pozzi G, Valensin PE, Galieni P, Bigazzi C. Recurrent Septicemia in an Immunocompromised Patient Due to Probiotic Strains of Bacillus subtilis. J. Clin. Microbiol. 1998;38(1):325-6.

22. Cesaro S, Chinello P, Rossi L, Zanesco L. *Saccharomyces cerevisiae* fungemia in a neutropenic patient treated with *Saccharomyces boulardii*. Support Care Cancer. 2000;8(6):504–5.

23. Lungarotti MS, Mezzetti D, Radicioni M. Methaemoglobinaemia with concurrent blood isolation of *Saccharomyces* and *Candida*. Arch Dis Child Educ Pract Ed. 2003;88(5):446.

24. Tommasi C, Equitani F, Masala M et al. Diagnostic difficulties of *Lactobacillus casei* bacteraemia in immunocompetent patients: A case report. J Med Case Rep. 2008;2(315):1-4.

25. Santino I, Alari A, Bono Set al. *Saccharomyces cerevisiae* fungemia, a possible consequence of the treatment of *Clostridium difficile* colitis with a probioticum. Int J Immunopathol Pharmacol. 2014;27(1):143-6.

26. Meini S, Laureano R, Fani L et al. Breakthrough *Lactobacillus rhamnosus GG* bacteremia associated with probiotic use in an adult patient with severe active ulcerative colitis: case report and review of the literature. Infection. 2015;43(6):777-81.

27. Richard V, Auwera PV, Snoeck R, Daneau D, Meunier F. Nosocomial bacteremia caused by *Bacillus* species. Eur. J. Clin. Microbiol. Infect. Dis. 1988;7(6):783-85.

28. Pletincx M, Legein J, Vandenplas Y. Fungemia with *Saccharomyces boulardii* in a 1-year-old girl with protracted diarrhea. J Pediatr Gastroenterol Nutr. 1995;21(1):113-5.

29. Rijnders BJA, Wijngaerden E, Verwaest C, Peetermans WE. *Saccharomyces* fungemia complicating *Saccharomyces boulardii* treatment in a nonimmunocompromised host. Intensive Care Med. 2000;26(6):825.

30. Cherifi S, Robberecht J, Miendje Y. *Saccharomyces cerevisiae* fungemia in an elderly patient with *Clostridium difficile* colitis. Acta Clin Belg. 2004;59(3):223-4.

31. Henry S, D’Hondt L, André M, Holemans X, Canon JL. *Saccharomyces cerevisiae* fungemia in a head and neck cancer patient: a case report and review of the literature. Acta Clin Belg. 2004;59(4):220-2.

32. Lestin F, Pertschy A, Rimek D. Fungemia after oral treatment with *Saccharomyces boulardii* in a patient with multiple comorbidities. Dtsch Med Wochenschr. 2003;128(48):2531-3.

33. Burkhardt O, Köhnlein T, Pletz MW, Welte T. *Saccharomyces boulardii* induced sepsis: successful therapy with voriconazole after treatment failure with fluconazole. Scand J Infect Dis. 2005;37(1):69-72.

34. Guenther K, Straube E, Pfister W, Guenther A, Huebler A. Sever sepsis after probiotic treatment with *Escherichia coli* NISSLE 1917. 2010;29(2):188-9.

35. Jenke A, Ruf EM, Hoppe T, Heldmann M, Wirth S. *Bifidobacterium* septicaemia in an extremely low-birthweight infant under probiotic therapy. Arch Dis Child Educ Pract Ed. 2012;97(3):217-8.

36. Bassetti S, Frei R, Zimmerli W. Fungemia with *Saccharomyces cerevisiae* after treatment with *Saccharomyces boulardii*. Am J Med. 1998;105(1):71-2.

37. Conen A, Zimmerer S, Frei R, Battegay M, Elzi L. A pain in the neck: probiotics for ulcerative colitis. Ann Intern Med. 2009;151(12):895-7.

38. Bertelli C, Pillonel T, Torregrossa A et l. *Bifidobacterium longum* bacteremia in preterm infants receiving probiotics. Clin Infect Dis. 2015;60(6):924-7.

39. Zbinden A, Zbinden R, Berger C, Arlettaz R. Case series of *Bifidobacterium longum* bacteremia in three preterm infants on probiotic therapy. Neonatology. 2015;107(1):56-9.

40. Lolis N, Veldekis D, Moraitou H et al. *Saccharomyces boulardii* fungaemia in an intensive care unit patient treated with caspofungin. Crit Care. 2008;12(2):414.

41. Stefanatou E, Kompoti M, ParidouA et al. Probiotic sepsis due to *Saccharomyces fungaemia* in a critically ill burn patient. Mycoses.2001;54(5):643-6.

42. Papanikolaou MN, Balla M, Papavasilopoulou T, Kofi G, Karatzas S. Probiotics: an obedient ally or an insidious enemy? Crit Care. 2012;16(6):456.

43. Perapoch J, Planes AM, Querol A et al. Fungemia with *Saccharomyces cerevisiae* in two newborns, only one of whom had been treated with ultra-levura. Clin Microbiol Infect. 2000;19(6):468-70.

44. Munõz P, Bouza E, Cuenca-Estrella M et al. *Saccharomyces cerevisiae* fungemia: an emerging infectious disease. Clin Infect Dis. 2005;40(1):1625-34.

45. Mackay A , Taylor M, Kibbler CC, Hamilfn JMT. *Lactobacillus* endocarditis caused by a probiotic organismo. Clin Microbiol Infect. 1999;5(5):290-2.

46. MacGregor G, Smith AJ, Thakker B, Kinsella J. Yoghurt biotherapy: contraindicated in immunosuppressed patients? Postgrad Med J. 2002;78:366-7.

47. Brecht M, Garg A, Longstaff K, Cooper C, Andersen C. *Lactobacillus* sepsis following a laparotomy in a preterm infant: A note of caution. Neonatology. 2016;109(3):186-9.

48. Presterl E, Kneifel W, Mayer H, Zehetgruber M, Makristathis A. Endocarditis by *Lactobacillus rhamnosus* due to yogurt ingestion? Scand J Infect Dis. 2001;33(9):710-4.

49. da Silva FHA, Paço FR, Reis E, Amaral V. Infecção por *Saccharomyces cerevisae* – uma infecção atípica em UTI. Rev Bras Ter Intensiva. 2011;23(1):108-111.

50. De Groote MA, Frank DN, Dowell E, Glode MP, Pace NR. *Lactobacillus rhamnosus GG* bacteremia associated with probiotic use in a child with short gut syndrome. Pediatr Infect Dis J. 2005;23(1):278:80.

51. Riquelme AJ, Calvo MA, Guzmán AM. *Saccharomyces cerevisiae* fungemia after *Saccharomyces boulardii* treatment in immunocompromised patients. J Clin Gastroenterol. 2006;36(1):41-3.

52. Thygesen JB, GlerupH, Tarp B. *Saccharomyces boulardii* fungemia caused by treatment with a probioticum. BMJ case reports. 2012;27:1-3.

53. Avcin SL, Pokorn MP, Kitanovski L, Premru MM, Jazbec J. *Bifidobacterium breve* Sepsis in Child with High-Risk Acute Lymphoblastic Leukemia. Emerg Infect Dis. 2015;21(9):1674-5.

54. Rautio M, Jousimies-Somer H, Kauma H et al. Liver abscess due to a *Lactobacillus rhamnosus* strain indistinguishable from *L. rhamnosus strain GG*. Clin Infect Dis. 1999;28(5):1159-60.

55. Ohishi A, Takahashi S, Ito Y et al. *Bifidobacterium* septicemia associated with postoperative probiotic therapy in a neonate with omphalocele. J. Pediatr. 2010;156(4):679-81.

56. Zein EF, Karaa S, Chemaly A et al. *Lactobacillus rhamnosus* septicemia in a diabetic patient associated with probiotic use: a case report. Ann Biol Clin (Paris). 2008;66(2):195-8.

57. Esaiassen E, Cavanagh P, Hjerde E et al. *Bifidobacterium longum* Subspecies infantis Bacteremia in 3 Extremely Preterm Infants Receiving Probiotics. Emerg Infect Dis. 2016;22(9):1664-6.

58. Kochan P, Chmielarczyk A, Szymaniak L et al. *Lactobacillus rhamnosus* administration causes sepsis in a cardiosurgical patient--is the time right to revise probiotic safety guidelines? Clin Microbiol Infect. 2011;17(10):1589-92.

59. Chioukh FZ, Hmida HB, Ameur KB, Toumi A. *Saccharomyces cerevisiae* fungemia in a premature neonate treated receiving probiotics. Med Mal Infect. 2013;43(8):359-60.

60. Eren Z, Gurol Y, Sonmezoglu M et al. *Saccharomyces cerevisiae* fungemia in an elderly patient following probiotic treatment. Mikrobiyol Bul. 2014;48(2):351-5.

61. Romanio MR, Coraine LA, Maielo VP, Abramczyc ML, Souza RL, Oliveira NF. *Saccharomyces cerevisiae* fungemia in a pediatric patient after treatment with probiotics. Rev Paul Pediatr. 2017;35(3):361-364.

62. Ujjwayini Roy, Laxman G. Jessani et al. Seven cases of *Saccharomyces* fungaemia related to use of probiotics. Mycoses 2017; 1–6. DOI: 10.1111/myc.12604

63. Martin Isabella W., Tonner Rita, Trivedi Julie, Miller Heather, Lee Richard, Liang Xinglun, Rotello Leo, Isenbergh Elena, Anderson Jennifer, Perl Trish, Zhang Sean X., *Saccharomyces boulardii* probiotic-associated fungemia: questioning the

safety of this preventive probiotic’s use, Diagnostic Microbiology and Infectious Disease (2016), doi: 10.1016/j.diagmicrobio.2016.12.004.

64. Kato K, Funabashi N, Takaoka H et al. *Lactobacillus paracasei* endocarditis in a consumer of probiotics with advanced and severe bicuspid aortic valve stenosis complicated with diffuse left ventricular mid-layer fibrosis. Int J Cardiol. 2016. http://dx.doi.org/10.1016/j.ijcard.2016.09.002.

65. Ellouze O, et al. Septic shock due to Saccharomyces boulardii. Med Mal Infect (2015),http://dx.doi.org/10.1016/j.medmal.2015.12.003.

66. Molinaro M, Aiazzi M, La Torre A et al. Sepsi da Lactobacillus Rhamnosus associato all’utilizzo di un integratore probiotico in un neonato pretermine: case report. Recenti Prog Med 2016; 107: 485-486.

67. Pararajasingam A, Uwagwu J. *Lactobacillus*: the not so friendly bacteria BMJ Case Rep Published Online: doi:10.1136/bcr-2016-218423.

**Table S1.** Cases of fungemia after use of probiotics in 35 patients identified by systematic review, 1976-2018.

| **Author, year** | **Country** | **NIH** | **Age, sex** | **Conditions associated** | **Indication of probiotic** | **Probiotic microorganisms isolated from biological samples** | **Method of comparison with probiotics** | **Death** |
| --- | --- | --- | --- | --- | --- | --- | --- | --- |
| Jensen, 1976 | USA | Fair | 68 y, M. | Obesity, SAH. | Self-medication | *Saccharomyces spp.* | Unrealized | No |
| Viggiano, 1995 | France | Fair | 14 y, M. | Large burnt, CA, EN. | Noninvasive diarrhea | *Saccharomyces cerevisiae* | Unrealized | No |
| Pletincx, 1995 | Belgium | Fair | 1 y, F. | Diarrhea, malnutrition, CA, PN. | Bacterial overgrowth | *Saccharomyces cerevisiae* | Unrealized | No |
| Bassetti, 1998 | Switzerland | Fair | 51 y, M. | Polyarteritis, immunosuppressed, CA, *C. difficile* colitis. | *C. difficile* colitis | *Saccharomyces cerevisiae* | Protein electrophoresis | No |
| Fredenucci, 1998 | France | Fair | 49 y, M. | Pneumonia, CA, EN. | Noninvasive diarrhea | *Saccharomyces cerevisiae* | Protein electrophoresis | No |
| Niault, 1999 | France | Fair | 78 y, F. | COPD, CA, EN. | Noninvasive diarrhea | *Saccharomyces cerevisiae* | Unrealized | No |
| Hennequin, 2000 | France | Fair | 2 y, M. | Cystic fibrosis, Ileum atresia, intestinal surgery, CA, PN. | Nutritional intolerance | *Saccharomyces boulardii* | Unrealized | No |
| Hennequin, 2000 | France | Fair | 36 y, M. | HIV, brain lymphoma, EN, CA. | Noninvasive diarrhea | *Saccharomyces boulardii* | Unrealized | No |
| Hennequin, 2000 | France | Fair | 47 y, M. | Esophagus adenocarcinoma, EN, CA. | Noninvasive diarrhea | *Saccharomyces boulardii* | Unrealized | No |
| Hennequin, 2000 | France | Fair | 72 y, F. | COPD, pneumonia. | EN supplement | *Saccharomyces boulardii* | Unrealized | No |
| Cesaro, 2000 | Italy | Fair | 9 m, NS. | ALL, neutropenia, CA. | To prevent AAD | *Saccharomyces cerevisiae* | Unrealized | No |
| Lherm, 2002 | France | Fair | 50 y, M. | Cardiac arrest, CA, EN. | NS | *Saccharomyces cerevisiae* | DNA polymorphism | Yes |
| Lherm, 2002 | France | Fair | 51 y, F. | Vascular surgery, cachexia, EN, CA. | NS | *Saccharomyces cerevisiae* | DNA polymorphism | Yes |
| Lherm, 2002 | France | Fair | 82 y, F. | Acute respiratory stress, CA, EN. | NS | *Saccharomyces cerevisiae* | DNA polymorphism | No |
| Lherm, 2002 | France | Fair | 75 y, M. | Acute respiratory stress, CA, EN. | NS | *Saccharomyces cerevisiae* | DNA polymorphism | No |
| Lherm, 2002 | France | Fair | 77 y, M. | Duodenal ulcer, peritonitis, CA, EN. | NS | *Saccharomyces cerevisiae* | DNA polymorphism | Yes |
| Lherm, 2002 | France | Fair | 71 y, F. | Hemorrhagic stroke, CA, EN. | NS | *Saccharomyces cerevisiae* | DNA polymorphism | No |
| Riquelme, 2003 | Chile | Fair | 42 y, F. | Solid organ transplantation, immunosuppressed, *C. difficile* colitis*.* | *C. difficile* colitis | *Saccharomyces cerevisiae* | Unrealized | No |
| Riquelme, 2003 | Chile | Fair | 41 y, M. | HIV, meningoencephalitis, CA. | Noninvasive diarrhea | *Saccharomyces cerevisiae* | Protein electrophoresis | No |
| Cherifi, 2004 | Belgium | Fair | 89 y, F. | Anorexia, gastrostomy, EN, *C. difficile* colitis*.* | *C. difficile* colitis | *Saccharomyces cerevisiae* | Unrealized | Yes |
| Henry, 2004 | Belgium | Fair | 65 y, M. | Oropharynx carcinoma, CA, PN. | Noninvasive diarrhea | *Saccharomyces cerevisiae* | Unrealized | No |
| Muñoz, 2005 | Espain | Good | 72 y, F. | Cardiac surgery, EN, *C. difficile* colitis*.* | *C. difficile* colitis | *Saccharomyces cerevisiae* | DNA amplification | Yes |
| Muñoz, 2005 | Espain | Good | 74 y, F. | Rheumatoid arthritis, cardiac surgery, *C. difficile* colitis*.* | *C. difficile* colitis | *Saccharomyces cerevisiae* | DNA amplification | Yes |
| Muñoz, 2005 | Espain | Good | 76 y, F. | Cardiac surgery, EN, CA, *C. difficile* colitis*.* | *C. difficile* colitis | *Saccharomyces cerevisiae* | DNA amplification | Yes |
| Lolis, 2008 | Greece | Fair | 56 y, M. | Septic shock, EN, CA. | Noninvasive diarrhea | *Saccharomyces cerevisiae* | DNA amplification | No |
| da Silva, 2011 | Brazil | Fair | 73 y, M. | COPD, bladder cancer, diarrhea. | Noninvasive diarrhea | *Saccharomyces cerevisiae* | Unrealized | Yes |
| Thygesen, 2012 | Denmark | Fair | 79 y, F. | Rheumatoid arthritis, EN, CA, *C. difficile* colitis*.* | *C. difficile* colitis | *Saccharomyces cerevisiae* | Manual microbiology | Yes |
| Martin, 2016 | USA | Fair | 60 y, M. | Septic shock, EN, CA. | To prevent AAD | *Saccharomyces cerevisiae* | DNA amplification | No |
| Romanio, 2017 | Brazil | Fair | 1y,M. | Severe chronic malnutrition, congenital cardiac disease correction | Nutritional intolerance | *Saccharomyces cerevisiae* | Unrealized | No |
| Roy, 2017 | India | Good | 7m, M. | Preterm, CA, PN. | To prevent NE | *Saccharomyces cerevisiae* | DNA amplification | Yes |
| Roy, 2017 | India | Good | 8m, M. | Preterm, neonatal sepsis. | To prevent NE | *Saccharomyces cerevisiae* | DNA amplification | No |
| Roy, 2017 | India | Good | 75y, M. | Respiratory failure, CA. | To prevent AAD | *Saccharomyces cerevisiae* | DNA amplification | No |
| Roy, 2017 | India | Good | 37 y, M. | Polytrauma, diarrhoea. | Noninvasive diarrhea | *Saccharomyces cerevisiae* | DNA amplification | No |
| Roy, 2017 | India | Good | 25 y, F. | Acute pancreatitis, PN. | To prevent AAD | *Saccharomyces cerevisiae* | DNA amplification | No |
| Roy, 2017 | India | Good | 66 y, F. | Cerebral stroke, multiple episodes of sepsis. | Noninvasive diarrhea | *Saccharomyces cerevisiae* | DNA amplification | No |

Source: elaborated by the author. AAD=Antimicrobial-associated diarrhea, ALL=Acute lymphocytic leukemia, CA=central access, COPD=chronic obstructive pulmonary disease, DNA=deoxyribonucleic acid, EN=Enteral nutrition, F. = female, HIV=human immunodeficiency vírus, M. = male, NS=Not Shown, PN=parenteral nutrition, SAH=systemic arterial hypertension, y=years.

**Table S2.** Sepsis cases after use of probiotics in 29 patients identified by systematic review, 1976-2018.

| **Author, year** | **Country** | **NIH** | **Age, sex** | **Conditions associated** | **Indication of probiotic** | **Probiotic microorganisms isolated from biological samples** | **Method of comparison with probiotics** | **Death** |
| --- | --- | --- | --- | --- | --- | --- | --- | --- |
| Boucaud, 1996 | France | Fair | 49 y, F. | Bronchoaspiration, EN, CA. | Noninvasive diarrhea | *Saccharomyces cerevisiae* | Protein electrophoresis | No |
| Oggioni, 1998 | Italy | Fair | 73 y, M. | Chronic lymphocytic leukemia | NS | *Bacillus subtilis* | DNA polymorphism | Yes |
| Rijnders, 2000 | Belgium | Fair | 74 y, M. | Neurosurgery, EN, CA. | Noninvasive diarrhea | *Saccharomyces cerevisiae* | Unrealized | Yes |
| Perapoch, 2000 | Spain | Fair | 3 m, M. | Cardiac surgery, diarrhea, PN, CA. | Noninvasive diarrhea | *Saccharomyces cerevisiae* | Mitochondrial DNA | No |
| MacGregor, 2002 | United Kingdom | Fair | 42 y, F. | Sjögren’s syndrome, chronic kidney disease, CA, *C. difficile* colitis*.* | *C. difficile* colitis | *Lactobacillus rhamnosus* | Unrealized | Yes |
| Lestin, 2003 | Germany | Fair | 48 y, M. | Chronic pancreatitis, diabetes, wet gangrene, EN, CA, *C. difficile* colitis*.* | *C. difficile* colitis | *Saccharomyces cerevisiae* | Unrealized | Yes |
| Lungarotti, 2003 | Italy | Fair | 27 d, M. | Preterm, PN, CA. | To prevent bacterial overgrowth | *Saccharomyces cerevisiae* | Unrealized | No |
| Burkhardt, 2005 | Germany | Fair | 19 y, M. | Brain abscess, EN. | NS | *Saccharomyces cerevisiae* | Unrealized | No |
| Land, 2005 | USA | Fair | 1 m, M. | Cardiac surgery, EN, CA. | AAD | *Lactobacillus spp.* | DNA amplification | No |
| Land, 2005 | USA | Fair | 6 y, F. | Microcephaly, gastrostomy, urinary tract infection, PN, CA. | AAD | *Lactobacillus spp.* | DNA amplification | No |
| De Groote, 2005 | Canada | Fair | 11 m, M. | Rotavirus diarrhea, EN, CA. | Rotavirus diarrhea | *Lactobacillus rhamnosus* | DNA amplification | No |
| Piechno, 2007 | France | Fair | 61 y, M. | Hemilaryngectomy, tumor, PN, CA, *C. difficile* colitis*.* | *C. difficile* colitis | *Saccharomyces cerevisiae* | Unrealized | No |
| Zein, 2008 | Libya | Fair | 54 y, F. | SAH, diabetes, hyphotyroidism. | Self-medication | *Lactobacillus rhamnosus* | Unrealized | No |
| Ohishi, 2010 | Japan | Fair | 10 d, F. | Surgery for omphalocele, EN, CA. | NS | *Bifidobacterium breve* | DNA polymorphism | No |
| Guenther, 2010 | Germany | Fair | 25 d, NI. | Preterm, rotavirus diarrhea, CA. | Rotavirus diarrhea | *Escherichia coli* | Protein electrophoresis | No |
| Kochan, 2011 | Poland | Fair | 24 y, F. | Cardiac surgery, CA. | To prevent AAD | *Lactobacillus rhamnosus* | DNA amplification | No |
| Stefanatou, 2011 | Greece | Fair | 34 y, F. | Large burnt, EN, CA. | Improve EN tolerance | *Saccharomyces cerevisiae* | DNA amplification | Yes |
| Papanikolaou, 2012 | Greece | Fair | 64 y, M. | Biliary pancreatitis, PN, CA. | Noninvasive diarrhea | *Pediococcus pentosaceus* | Unrealized | No |
| Jenke, 2012 | Germany | Fair | 18 d, F. | Preterm, EN, CA. | To prevent NE | *Bifidobacterium infantis* | PCR | No |
| Metha, 2013 | USA | Fair | 69 y, M. | Lymphoma, CA. | Self-medication | *Lactobacillus acidophilus* | Unrealized | No |
| Chioukh, 2013 | Tunisia | Fair | 17 d, M. | Preterm, hyaline membrane disease, EN, PN, CA. | To prevent NE | *Saccharomyces cerevisiae* | Unrealized | No |
| Eren, 2014 | Turkey | Fair | 88 y, F. | SAH, urinary sepsis, CA. | Noninvasive diarrhea | *Saccharomyces cerevisiae* | Unrealized | No |
| Santino, 2014 | Italy | Fair | 86 y, M. | COPD, CA, *C. difficile* colitis*.* | *C. difficile* colitis | *Saccharomyces cerevisiae* | RNA amplification | No |
| Avcin, 2015 | Slovenia | Fair | 2 y, M. | Acute lymphocytic leukemia, CA. | NS | *Bifidobacterium breve* | Unrealized | No |
| Bertelli, 2015 | Switzerland | Good | 14 d, F. | Preterm, EN, CA. | To prevent NE | *Bifidobacterium infantis* | DNA polymorphism | No |
| Bertelli, 2015 | Switzerland | Good | 10 d, F. | Preterm, EN. | To prevent NE | *Bifidobacterium infantis* | DNA polymorphism | No |
| Ellouze, 2015 | France | Fair | 31 y, F. | Cardiac transplantation, CA, EN. | Noninvasive diarrhea | *Saccharomyces cerevisiae* | Unrealized | Yes |
| Brecht, 2016 | Australia | Fair | 2 m, M. | Laparotomy with ileostomy for ileal perforation, EN, CA. | NS | *Lactobacillus rhamnosus* | RNA amplification | No |
| Molinaro, 2016 | Italy | Fair | 5m, M. | Preterm. | To prevent NE | *Lactobacillus rhamnosus* | Unrealized | No |

Source: elaborated by the author. AAD=Antimicrobial-associated diarrhea, CA=central access, COPD= chronic obstructive pulmonary disease, d=days, DNA=deoxyribonucleic acid, EN=enteral nutrition, F.=female, M=male, m=months, NE=necrotizing enterocolitis, NS=not shown, PCR= Polymerase chain reaction, PN=parenteral nutrition, RNA=ribonucleic acid, SAH= systemic arterial hypertension, y=years.

**Table S3.** Cases of bacteremia after use of probiotics in 19 patients identified by systematic review, 1976-2018.

| **Author, year** | **Country** | **NIH** | **Age, sex** | **Conditions associated** | **Indication of probiotic** | **Probiotic microorganisms isolated from biological samples** | **Method of comparison with probiotics** | **Death** |
| --- | --- | --- | --- | --- | --- | --- | --- | --- |
| Richard V, 1988 | Belgium | Good | 47 y, M. | Head trauma, EN. | Noninvasive diarrhea | *Bacillus subtilis* | Antimicrobial susceptibiliy test | Yes |
| Richard V, 1988 | Belgium | Good | 25 y, M. | Head trauma, EN. | Noninvasive diarrhea | *Bacillus subtilis* | Antimicrobial susceptibiliy test | No |
| Richard V, 1988 | Belgium | Good | 63 y, F. | Endometrial carcinoma, EN. | Noninvasive diarrhea | *Bacillus subtilis* | Antimicrobial susceptibiliy test | No |
| Richard V, 1988 | Belgium | Good | 79 y, F. | Stroke, EN. | Noninvasive diarrhea | *Bacillus subtilis* | Antimicrobial susceptibiliy test | Yes |
| Barton, 2001 | USA | Fair | 3 m, F. | Intestinal surgery, EN, preterm. | Reestablish intestinal microbiota | *Pediococcus spp.** | Unrealized | No |
| Kunz, 2004 | USA | Good | 6 m, M. | Intestinal surgery, EN, PN. | Not shown | *Lactobacillus spp.* | DNA amplification | No |
| Kunz, 2004 | USA | Good | 4 m, M. | Intestinal surgery, EN, PN, CA. | To prevent bacterial overgrowth | *Lactobacillus spp.* | Unrealized | No |
| LeDoux, 2006 | USA | Fair | 38 y, M. | HIV, Hodgkin’s lymphoma, CA. | Not shown | *Lactobacillus acidophilus* | Unrealized | No |
| Tommasi, 2008 | Italy | Fair | 66 y, M. | COPD, diverticular disease. | Self-medication | *Lactobacillus casei* | Unrealized | No |
| Vahabnezhad, 2013 | USA | Fair | 17 y, M. | Ulcerative colitis, immunosuppressed | Self-medication | *Lactobacillus rhamnosus* | RNA amplification | No |
| Zbinden, 2015 | Switzerland | Good | 20 d, F. | Preterm, EN, CA. | To prevent NE | *Bifidobacterium longum* | RNA amplification | No |
| Meini, 2015 | Italy | Fair | 64 y, F. | Ulcerative colitis, immunosuppressed | Reestablish intestinal microbiota | *Lactobacillus rhamnosus* | Protein electrophoresis | No |
| Zbinden, 2015 | Switzerland | Good | 20 d, M. | Preterm, pulmonar dysplasia, EN, CA. | To prevent NE | *Bifidobacterium longum* | RNA amplification | No |
| Haghighat, 2015 | USA | Fair | 51 y, M. | HIV, hepatical cirrhosis. | Self-medication | *Lactobacillus acidophilus* | Unrealized | No |
| Zbinden, 2015 | Switzerland | Good | 11 d, F. | Preterm, EN. | To prevent NE | *Bifidobacterium longum* | RNA amplification | No |
| Esaiassen, 2016 | Norway | Good | 12 d, M. | ARDS, EN, CA. | To prevent NE | *Bifidobacterium longum* | Nucleotide-level variation | No |
| Esaiassen, 2016 | Norway | Good | 12 d, M. | ARDS, EN, CA. | To prevent NE | *Bifidobacterium longum* | Nucleotide-level variation | No |
| Esaiassen, 2016 | Norway | Good | 46 d, F. | ARDS, EN, CA. | To prevent NE | *Bifidobacterium longum* | Nucleotide-level variation | No |
| Aroutcheva, 2016 | USA | Fair | 69 y, M. | Heart failure, ischemic colitis, EN, CA. | To prevent AAD | *Lactobacillus acidophilus* | DNA amplification** | No |

Source: elaborated by the author. AAD=Antimicrobial-associated diarrhea, ARDS= acute respiratory distress syndrome, CA=central access, COPD= chronic obstructive pulmonary disease, d=days, DNA=deoxyribonucleic acid, EN=enteral nutrition, F.=female, HIV=human immunodeficiency vírus, M=male, m=months, NE=necrotizing enterocolitis, NS=not shown, PN=parenteral nutrition, RNA=ribonucleic acid, y=years.

*Gender differs from probiotic; ** microorganism was considered unrelated to probiotic.

**Table S4.** Cases of endocarditis after using probiotics in 4 patients identified by systematic review, 1976-2018.

| **Author, year** | **Country** | **NIH** | **Age, sex** | **Conditions associated** | **Indication of probiotic** | **Probiotic microorganisms isolated from biological samples** | **Method of comparison with probiotics** | **Death** |
| --- | --- | --- | --- | --- | --- | --- | --- | --- |
| Mackay, 1999 | United Kingdom | Fair | 67 y, M. | Mitral valve prolapse, dental extraction. | Self-medication | *Lactobacillus rhamnosus* | Mass spectrometry | No |
| Presterl, 2001 | Áustria | Fair | 23 y, M. | Bicuspid aortic valve. | Self-medication | *Lactobacillus rhamnosus* | Unrealized | No |
| Franko, 2013 | France | Fair | 77 y, M. | Prostate tumor, mitral valve prolapse, colonoscopy. | Self-medication | *Lactobacillus paracasei* | Unrealized | No |
| Kato, 2016 | Japão | Fair | 78 y, M. | Elderly. | Self-medication | *Lactobacillus paracasei* | Unrealized | No |

Source: elaborated by the author.

**Table S5.** Cases of abscess, empyema, septic arthritis and pneumonia after use of probiotics in 6 patients identified by systematic review, 1976-2018.

| **Author, year** | **Country** | **NIH** | **Age, sex** | **Conditions associated** | **Indication of probiotic** | **Probiotic microorganisms isolated from biological samples** | **Diagnostic** | **Method of comparison with probiotics** | **Death** |
| --- | --- | --- | --- | --- | --- | --- | --- | --- | --- |
| Rautio, 1999 | Finland | Fair | 74 y, F. | SAH, diabetes. | Self-medication | *Lactobacillus rhamnosus* | Hepatic abscess | PCR | No |
| Conen, 2009 | Switzerland | Fair | 38 y, F. | Ulcerative colitis, immunosuppressed. | Inflammatory bowel disease | *Lactobacillus rhamnosus* | Epidural and retropharyngeal abscess | Gene Sequencing | No |
| Luong, 2010 | USA | Fair | 56 y, M. | HIV, solid organ transplantation. | *C. difficile* colitis | *Lactobacillus rhamnosus* | Pleural empyema | RNA amplification | No |
| Bush, 2014 | USA | Fair | 52 y, F. | Hip arthroplasty. | Self-medication | *Bifidobacterium spp.* | Septic arthritis | Unrealized | No |
| Doern, 2014 | USA | Fair | 11 m, F. | Esophageal surgery, EN. | Not shown | *Lactobacillus rhamnosus* | Pneumonia | PCR | No |
| Pararajasingam, 2017 | Reino Unido | Fair | 65 y, F. | Hypertension, diabetes mellitus. | Self-medication | *Lactobacillus paracasei* | Hepatic abscess | RNA amplification | No |

Source: elaborated by the author. EN=enteral nutrition, F.=female, HIV= human immunideficiency vírus, M=male, m=months, NS=not shown, PCR= Polymerase chain reaction, RNA=ribonucleic acid, SAH= systemic arterial hypertension, y=years.

**Table S6.** Antimicrobial susceptibility test standard of Saccharomyces spp. of patients with infectious complication following probiotic use, identified by systematic review, 1976-2018.

|  |  |  | **Group of antifungals** | | |
| --- | --- | --- | --- | --- | --- |
| Fungi | Total | Pattern | Azoles | Anphotericin | Echinocandins |
| *Saccharomyces spp.* | 12 | S | 10 | 8 | 8 |
|  |  | R | 2 | 2 |  |

Source: elaborated by the author. R=resistant, S=sensitive.

**Table S7.** Antimicrobial susceptibility test standard for isolates of probiotic-related microorganisms from patients with infectious complications following probiotic use, identified by systematic review, 1976-2018.

|  |  |  | **Antimicrobial groups** | | | | | | | | |
| --- | --- | --- | --- | --- | --- | --- | --- | --- | --- | --- | --- |
| Bacteria isolated | Total | Patterns | Penicillins + cephalosporins | BLC/BLI | Carbapenems | Linco | Aminog | Glycop | Dapto | SMT | Rifampicin |
| *Lactobacillus spp.* | 14 | S | 12 | 1 | 1 | 7 | 4 | 2 |  | 1 | 2 |
|  |  | R | 2 |  | 1 |  |  | 8 |  | 1 |  |
| *Bifidobacterium spp.* | 5 | S | 5 | 2 | 1 | 1 |  |  |  |  |  |
|  |  | R |  |  | 1 |  | 1 |  |  |  |  |
| *Bacillus spp.* | 1 | S | 1 |  |  |  | 1 | 1 |  |  |  |
|  |  | R | 1 |  |  |  |  |  |  |  | 1 |
| *Pediococcus spp.* | 2 | S | 1 |  |  | 1 |  |  | 1 |  |  |
|  |  | R | 1 |  |  |  |  | 1 |  |  |  |

Source: elaborated by the author. Aminog=aminoglycosides, BLC/BLI= betalactam and beta-lactamase inhibitors, Dapto=daptomicina,Glicop=glicopetides, Linco=lincosamides, SMT= sulfamethoxazole and trimethoprim.

**Table S8**. Patients younger than 1 year with infectious complication following probiotic use, identified by systematic review, 1976-2018.

| **Author, year** | **Country** | **Age, sex** | **Preterm weight** | **Morbidity** | **Indication*** | **Diagnostic** | **Microorganism** |
| --- | --- | --- | --- | --- | --- | --- | --- |
| Cesaro, 2000 | Italy | 8 m, NS | No, NS. | Acute myeloid leukemia, chemotherapy, neutropenia | To prevent AAD | Fungemia | *S. cerevisiae* |
| Perapoch, 2000 | Spain | 3 m, M. | No, NS. | Congenital cardiopathy, chronic diarrhea | Diarrhea | Sepsis | *S. cerevisiae* |
| Barton, 2001 | USA | 3 m, F. | Yes, 2,108g | Gastroschisis, intestinal resection | Restore intestinal microbiota | Bacteremia | *Pediococcus sp.* |
| Lungarotti, 2003 | Italy | 27 d, M. | Yes, NS. | Food intolerance | To prevent bacterial overgrowth | Sepsis | *S. cerevisiae* |
| Kunz, 2004 | USA | 4 m, M. | No, NS. | Intestinal atresia, surgery, short bowel | NS | Bacteremia | *Lactobacillus spp* |
| Kunz, 2004 | USA | 6 m, M. | No, NS. | Gastroschisis, intestinal surgery | To prevent bacterial overgrowth | Bacteremia | *Lactobacillus spp* |
| Land, 2005 | USA | 1 m, M. | No, 3,200g | Post cardiac surgery, AAD | To treat AAD | Sepsis | *Lactobacillus spp* |
| De Groote, 2005 | Canada | 8 m, M. | No, NS. | NE, intestinal resection, short bowel | Rotavirus diarrhea | Sepsis | *L. rhamnosus* |
| Guenther, 2010 | Germany | 25 d, NS. | Yes, 935g | Rotavirus diarrhea | Restore intestinal microbiota | Sepsis | *E. coli NISSLE 1917* |
| Ohishi, 2010 | Japan | 10 d, F. | No, 2,060g | Omphalocele, intestinal resection | NS | Sepsis | *B. breve BBG-01* |
| Jenke, 2012 | Germany | 18 d, F. | Yes, 600g | Fetofetal transfusion syndrome | To prevent NE | Sepsis | *B. infantis* |
| Chioukh, 2013 | Tunisia | 17 d, M. | Yes, 950g | Hyaline membrane syndrome | To prevent NE | Sepsis | *S. cerevisiae* |
| Doern, 2014 | USA | 11 m, F. | No, NS. | Esophagus atresia, surgery, dysphagia | NS | Pneumonia | *L. rhamnosus* |
| Zbinden, 2015 | Switzerland | 20 d, F. | Yes, 1,200g | ARDS | To prevent NE | Bacteremia | *B. longum* |
| Zbinden, 2015 | Switzerland | 20 d, M. | Yes, 850g | Bronchopulmonary dysplasia | To prevent NE | Bacteremia | *B. longum* |
| Zbinden, 2015 | Switzerland | 11 d, F. | Yes, 1230g | ARDS | To prevent NE** | Bacteremia | *B. longum* |
| Bertelli, 2015 | USA | 14 d, F. | Yes, 867g | ARDS | To prevent NE | Sepsis | *B. infantis* |
| Bertelli, 2015 | USA | 10 d, F. | Yes, 1,090g | ARDS | To prevent NE** | Sepsis | *B. infantis* |
| Brecht, 2016 | Australia | 2 m, M. | Yes, 970g | Intestinal perforation | NS | Sepsis | *L. rhamnosus* |
| Esaiassen, 2016 | Norway | 12 d, M. | Yes, 730g | ARDS | To prevent NE** | Bacteremia | *B. longum* |
| Esaiassen, 2016 | Norway | 12 d, M. | Yes, 500g | ARDS | To prevent NE | Bacteremia | *B. longum* |
| Esaiassen, 2016 | Norway | 46 d, F. | Yes, 697g | ARDS | To prevent NE** | Bacteremia | *B. longum* |
| Molinaro, 2016 | Italy | 5 , M. | Yes, NS. | Preterm. | To prevent NE | Sepsis | *L. rhamnosus* |
| Roy, 2017 | Indian | 7 m, M. | Yes, 825g. | Preterm, CA, PN. | To prevent NE | Fungemia | *S. cerevisiae* |
| Roy, 2017 | Indian | 8 m, M. | Yes, 1500g. | Preterm, neonatal sepsis. | To prevent NE | Fungemia | *S. cerevisiae* |

Source: elaborated by the author. AAD=Antimicrobial-associated diarrhea, ARDS= acute respiratory distress syndrome, d=days, F.=female, M=male, m=months, NE=necrotizing enterocolitis, NS=not shown. * Indication of use of probiotic; ** Developed NE.
